# Hospitalisation rates and predictors in people with dementia: a systematic review and meta-analysis

**DOI:** 10.1186/s12916-019-1369-7

**Published:** 2019-07-15

**Authors:** Hilary Shepherd, Gill Livingston, Justin Chan, Andrew Sommerlad

**Affiliations:** 1 Division of Psychiatry, University College London, 6th Floor, Maple House, 149 Tottenham Court Road, London, W1T 7NF UK; 2 Camden and Islington NHS Foundation Trust, 4 St Pancras Way, London, NW1 0PE UK

**Keywords:** Hospitalisation, Dementia, Healthcare utilisation, Risk factors, Prognosis

## Abstract

**Background:**

Hospitalisation is often harmful for people with dementia and results in high societal costs, so avoidance of unnecessary admissions is a global priority. However, no intervention has yet reduced admissions of community-dwelling people with dementia. We therefore aimed to examine hospitalisation rates of people with dementia and whether these differ from people without dementia and to identify socio-demographic and clinical predictors of hospitalisation.

**Methods:**

We searched MEDLINE, Embase, and PsycINFO from inception to 9 May 2019. We included observational studies which (1) examined community-dwelling people with dementia of any age or dementia subtype, (2) diagnosed dementia using validated diagnostic criteria, and (3) examined all-cause general (i.e. non-psychiatric) hospital admissions. Two authors screened abstracts for inclusion and independently extracted data and assessed included studies for risk of bias. Three authors graded evidence strength using Cochrane’s GRADE approach, including assessing for evidence of publication bias using Begg’s test. We used random effects meta-analysis to pool estimates for hospitalisation risk in people with and without dementia.

**Results:**

We included 34 studies of 277,432 people with dementia: 17 from the USA, 15 from Europe, and 2 from Asia. The pooled relative risk of hospitalisation for people with dementia compared to those without was 1.42 (95% confidence interval 1.21, 1.66) in studies adjusted for age, sex, and physical comorbidity. Hospitalisation rates in people with dementia were between 0.37 and 1.26/person-year in high-quality studies. There was strong evidence that admission is associated with older age, and moderately strong evidence that multimorbidity, polypharmacy, and lower functional ability are associated with admission. There was strong evidence that dementia severity alone is not associated.

**Conclusions:**

People with dementia are more frequently admitted to hospital than those without dementia, independent of physical comorbidities. Future interventions to reduce unnecessary hospitalisations should target potentially modifiable factors, such as polypharmacy and functional ability, in high-risk populations.

**Electronic supplementary material:**

The online version of this article (10.1186/s12916-019-1369-7) contains supplementary material, which is available to authorized users.

## Background

The number of older people is expected to rise throughout the century, with the number of people with dementia rising in parallel [1]. Hospital admissions can be harmful and distressing for people with dementia who are less likely to receive adequate pain relief [2], more likely to receive potentially harmful medication [3], have a higher risk of delirium than those without dementia [4], and commonly decline functionally during admission [5]. Avoidable admissions are more frequent for people with dementia [6] and readmission is common [7]. Admissions are costlier for people with dementia than those without [8], and pressures on hospitals to reduce admission length could also mean that people with dementia are prematurely discharged from hospital into long-term care [9].

In this systematic review, we aimed to examine the rates of all general hospitalisations of people with dementia, whether these differ from those without dementia, and to identify socio-demographic and clinical predictors of admission. Understanding these would help to plan services for future increasing numbers of people with dementia. Previously designed interventions to reduce hospitalisations in people with dementia have not been found to be effective [10]. Identifying admission predictors may elucidate modifiable risk factors which future interventions could target in populations most likely to benefit.

### Aims

The following are the aims of the study:

- To compare hospitalisation risk in people with dementia to people without dementia
- To identify general hospital admission rates of people with dementia
- To examine demographic or clinical factors associated with higher hospital admission risk

## Methods

We prospectively registered the study protocol with PROSPERO (registration number CRD42018091722) (Additional file 2).

### Search strategy

We searched MEDLINE, Embase, and PsycINFO, including grey literature. The initial search was completed on 18 April 2018 and updated on 22 October 2018 and 9 May 2019 with no date or language restrictions. The search terms related to people with dementia, hospitalisation, and observational studies, using the SIGN filters for observational studies; the full search strategy is in Additional file 1: Table S1. We hand-searched included papers’ reference lists and contacted experts in the field to ensure a comprehensive review.

### Inclusion and exclusion criteria

We included observational studies which:

- Examined populations of people with dementia (any age or dementia subtype)
- Included clinical, research, or register populations of people with dementia, diagnosed using validated diagnostic criteria, e.g. International Statistical Classification of Diseases and Related Health Problems 10th revision, Diagnostic and Statistical Manual of Mental Disorders 5th edition, National Institute of Neurological and Communicative Disorders and Stroke, and the Alzheimer’s Disease and Related Disorders criteria
- Examined all-cause general (i.e. non-psychiatric) hospital admissions, providing sufficient data to examine the hospitalisation rates or potential risk factors

We excluded papers which:

- Examined populations who predominantly resided in long-term nursing care facilities
- Did not adequately delineate dementia, e.g. defined dementia by cognitive test score cut-off or solely by acetylcholinesterase inhibitor prescription. We included studies which supplemented register-derived dementia diagnosis by using medication prescription as an additional marker of dementia status
- Studied general populations of older people when it was impossible to separate out those with dementia
- Recruited study participants upon hospitalisation, as this prevents identification of factors leading to hospitalisation

### Screening papers

After excluding duplicate papers, one researcher (HS) screened the titles and abstracts using the eligibility criteria. A random 10% sample was independently reviewed for inclusion by a second researcher (JC); initial concordance regarding the study inclusion was 87%, and disagreements were resolved by a discussion with a third researcher (AS). Consensus on the inclusion of all studies was agreed by two researchers (HS, AS) with any disagreements resolved in a discussion with a third (GL).

### Data extraction and synthesis

Where available, the following information was extracted from included studies using a pre-piloted data extraction form (Additional file 1: Table S2): author, year and country of setting, participant number, mean age, sex distribution, method of diagnosis, study length and average follow-up time, average severity of dementia. Extracted outcome data were adjusted relative risk of hospitalisation in people with dementia compared to people without dementia and covariates included in fully adjusted models; percentage or rate of hospitalisation in people with dementia; socio-demographic or clinical factors examined as potential predictors of hospital admission; classification of factors and relative risk for their association with admission and covariates included in relative risk models. Two authors (HS and AS) independently extracted the outcome data.

We extracted data on admissions where the rate (per person-years (py)) or percentage of participants admitted to hospital was presented. Where the rate or proportion admitted was not provided, but raw data was available, we calculated the hospitalisation rate (number of hospitalisations/py) and/or percentage of participants admitted during study follow-up.

We prioritised obtaining the risk estimates provided for all-cause hospitalisation and, where this was not available, for admissions defined as preventable or ambulatory care sensitive conditions (ACSC), or for emergency rather than elective hospital admissions. Admissions caused by, for example, angina, infection, dehydration, or diabetes are deemed potentially avoidable, as proactive community care could prevent the need for a hospital stay [11, 12]. For studies with unclear data, we requested clarification directly from the study authors and included them in our primary analyses if this was received.

## Data analysis

### Quality assessment and analysis

Two researchers (HS, AS) assessed the risk of bias in individual papers using a modified version of the Newcastle-Ottawa Scale (NOS) for assessing the quality of cohort studies [13]. This considered definition of exposure, method of outcome ascertainment, selection and measurement bias, and confounding. Concordance on quality rating criteria was 89%, and consensus was reached through discussion in cases of disagreement on individual rating criteria. Full details of ratings are in Additional file 1: Table S3.

### Evidence grading

We then used the Cochrane Collaboration’s Grading of Recommendations, Assessment, Development and Evaluation (GRADE) approach to rate the confidence in the estimates given [14]. Grades of high, moderate, low, or very low were allocated based on the research team’s (AS, HS, GL) assessment of seven criteria: (1) risk of bias in individual studies assessed using NOS, (2) inconsistency (unexplained heterogeneity of results or non-overlapping 95% confidence intervals across studies), (3) indirectness (limited generalisability of the study populations), (4) imprecision (number of study participants and confidence intervals (CI) around the effect), (5) evidence of publication bias, (6) large magnitude of effect, and (7) evidence of dose-response relationship. Strength of evidence was graded down if factors 1–5 were noted and graded up if factor 6 or 7 was seen. To rate the precision, we performed a power calculation to find the number of participants needed to find a hospitalisation rate of 37%, as 0.37 hospitalisations/py was the lowest rate extracted from a paper at low risk of bias according to NOS, with 80% power and a significance level of 5%. Consequently, studies comprising > 349 participants with dementia were classed as precise. To rate the publication bias, we used Begg’s test when three or more studies measured an exposure in the same way, with *p* ≤ 0.10 indicating risk of publication bias [15], using STATA 14.

### Statistical analysis

We provide a narrative synthesis of findings from the included studies. For estimates of the risk of hospitalisation for people with dementia compared to those without in studies which adjusted for at least age, sex, and physical comorbidity, we used random effects meta-analysis to pool the results. Random effects models are appropriate where there is potential heterogeneity in study populations [16] and allows a combination of different measures of relative risk, e.g. hazard ratio and odds ratio [17]. We measured heterogeneity between studies using the *I*^2^ statistic and considered a priori that *I*^2^ > 50% indicated substantial heterogeneity. We judged we were unable to meta-analyse the findings on predictors of hospital admission as these frequently used heterogeneous exposure measures.

## Results

**Fig. 1:**
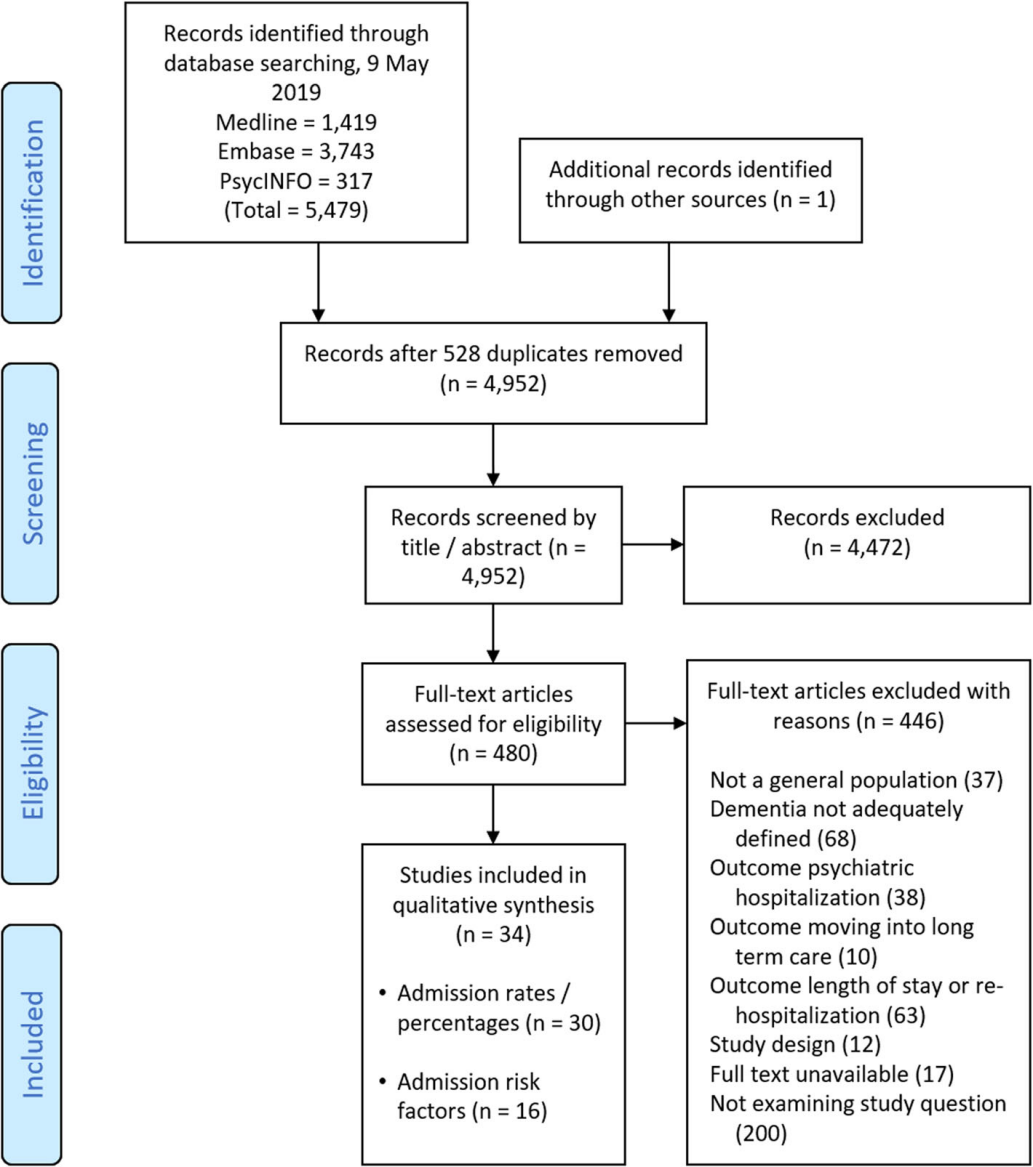
PRISMA flow diagram of included studies

We found 34 papers including a total of 277,432 people with dementia. The Preferred Reporting Items for Systematic Review and Meta-Analysis (PRISMA) diagram (Fig. 1) describes the results of the search and reasons for excluding studies [18]. Thirty-four included papers reported data from 1991 to 2016 and comprised 17 from the USA, five from England, three from France, three from Sweden, two from Finland, and one each from Taiwan, Germany, Hong Kong, and Scotland (Table 1). Five papers studied sub-populations: two were people with dementia at end of life, and one each of people with dementia and cancer, people with dementia and intellectual disability, and people with dementia and dysphagia, and we considered these separately.

**Table 1:** Description of 34 studies retrieved from systematic search

Author, publication year	Country	Years of data collection	Source of study participants	Number of people with dementia	Dementia type	Outcome ascertainment	Mean years follow-up (max)
Albert, 1999 [19]	USA	1996–97	Research cohort (WHICAP)	400	AD	Records and self/carer-report	1.75
Aupperle, 1999 [20]	USA	1997	Clinical sample	58	AD	Carer-report	1
Browne, 2017 [21]	England	2008–09	National register (CPRD)	4999	All-cause dementia	National register	3 (5)
Brüggenjürgen, 2015 [22]	Germany	2005–08	Insurance register (Statutory Health Insurance)	20,000	AD	Insurance register	4
Bynum, 2004 [23]	USA	1999	Insurance register (Medicare)	103,512	All-cause dementia	Insurance register	1
Callahan, 2012 [24]	USA	2001–08	Insurance registers (Medicare and Medicaid claims, MDS and OASIS)	1523	All-cause dementia	Insurance register	5.2
Chen, 2014 [25]	England	2008–10	National registers (CPRD and HES)	3896	AD	National register	2
Cortes, 2008 [26]	France	Not reported	Research cohort (REAL-FR)	686	Mild-moderate AD	Self-report	2
Hill, 2005 [27]	USA	1999–2002	Insurance register (Medicare)	3357	AD and VaD	Insurance register	3
Jennings, 2019 [28]	USA	2012–15	Insurance register (Medicare)	3249	All-cause dementia	Insurance register	1.75
Kunik, 2003 [29]	USA	1997–99	Insurance register (VAMC)	864	All-cause dementia	Insurance register	1.8 (2)
Malone, 2009 [30]	USA	2000–06	National register (HealthCore)	5396	AD	National register	2 (6)
Maust 2017 [31]	USA	2002–08	Research cohort (ADAMS)	332	All-cause dementia	Insurance register	6
Miu, 2018 [32]	Hong Kong	2013–15	Clinical sample	267	All-cause dementia	Hospital records	1 (1)
Mueller, 2017 [33]	England	2006–13	Clinical register (CRIS)	970	AD and DLB	National register	0.9
Mueller, 2018 [34]	England	2011–13	Clinical register (CRIS)	4668	All-cause dementia	National register	2
Nourhashemi, 2005 [35]	France	Not reported	Research cohort (REAL-FR)	677	AD	Self/carer-report	1
Phelan, 2012 [36]	USA	1994–2007	Research cohort (ACT)	494	All-cause dementia	Hospital records	9.6 (12)
Pimouguet, 2016 [11]	Sweden	2001–10	Research cohort (SNAC-K)	175	All-cause dementia	National register	4.7 (6)
Rudolph, 2010 [37]	USA	1991–2006	Research cohort (MADRC)	827	AD	Self/carer-report	3
Russ, 2015 [38]	Scotland	Not reported	National register (SDRIR)	730	All-cause dementia	National register	1.2 (3.3)
Sköldunger, 2015 [39]	Sweden	2001–04	Research cohort (SNAC)	319	All-cause dementia	Self/carer-report	3
Sloane, 2017 [40]	USA	Not reported	Clinical sample	136	AD	Carer-report	0.5
Sommerlad, 2019 [41]	UK	2008–16	Clinical register (CRIS)	10,137	All-cause dementia	Hospital records	2.5 (8.2)
Soto, 2015 [42]	France	Not reported	Research cohort (PLASA)	1131	AD	Self/carer-report	2
Thorpe, 2010 [12]	USA	1997–98	Research cohort (NLCS)	1186	AD and VaD	Insurance register	1
Tolppanen, 2015 [43]	Finland	2005–09	National register (Medication and Alzheimer’s disease study)	27,948	AD	National register	4 (2.2 to 4)
Zhao, 2008 [44]	USA	2003–04	Insurance register (Medicare)	25,109	All-cause dementia	Insurance register	1
Zhu, 2015 [45]	USA	1999–2010	Research cohort (WHICAP)	171	All-cause dementia	Insurance register	3.8 (10)
Studies including sub-populations of people with dementia					Special characteristics		
Axmon, 2016 [46]	Sweden	2002–12	National register (Swedish National Patient registry)	271	All-cause dementia (including 199 with ID)	National register	11
Chen, 2017 [47]	Taiwan	2002–11	Insurance register (National Health Insurance Research Database)	908	All-cause dementia during last year of life	Insurance register	9
Forma, 2011 [48]	Finland	1998–2003	National clinical registers	34,232	All-cause dementia during last 2 years of life	National register	2
Kedia, 2017 [49]	USA	2009	Insurance registers (Medicare and Medicaid)	9827	All-cause (including 1294 with comorbid cancer)	Insurance register	1
Tian, 2013 [50]	USA	2006–10	Insurance registers (MarketScans Commercial Claims and Medicare)	8977	AD (including 485 with dysphagia)	Insurance register	2 (3)

*ACT* adult changes in thought, *AD* Alzheimer’s disease, *ADAMS* Ageing, Demographics and Memory Study, *CRIS* Clinical Record Interactive Search, *DLB* dementia with Lewy bodies, *ID* intellectual disability, *MADRC* Massachusetts Alzheimer’s Disease Research Center, *MDS* minimum data set, *NLCS* National Longitudinal Caregiver Study, *NPI* Neuropsychiatric Inventory, *OASIS* Outcome And Assessment Information Set, *PLASA* Plan de Soin et d’Aide dans la maladie d’Alzheimer, *REAl-FR* French Network on Alzheimer’s Disease, *SDRIR* Scottish Dementia Research Interest Register, *SNAC(-K)* Swedish National Study on Aging and Care (-Kungsholmen), *VaD* vascular dementia, *WHICAP* Washington Heights-Inwood Columbia Aging Project

### Comparison to people without dementia

**Fig. 2:**
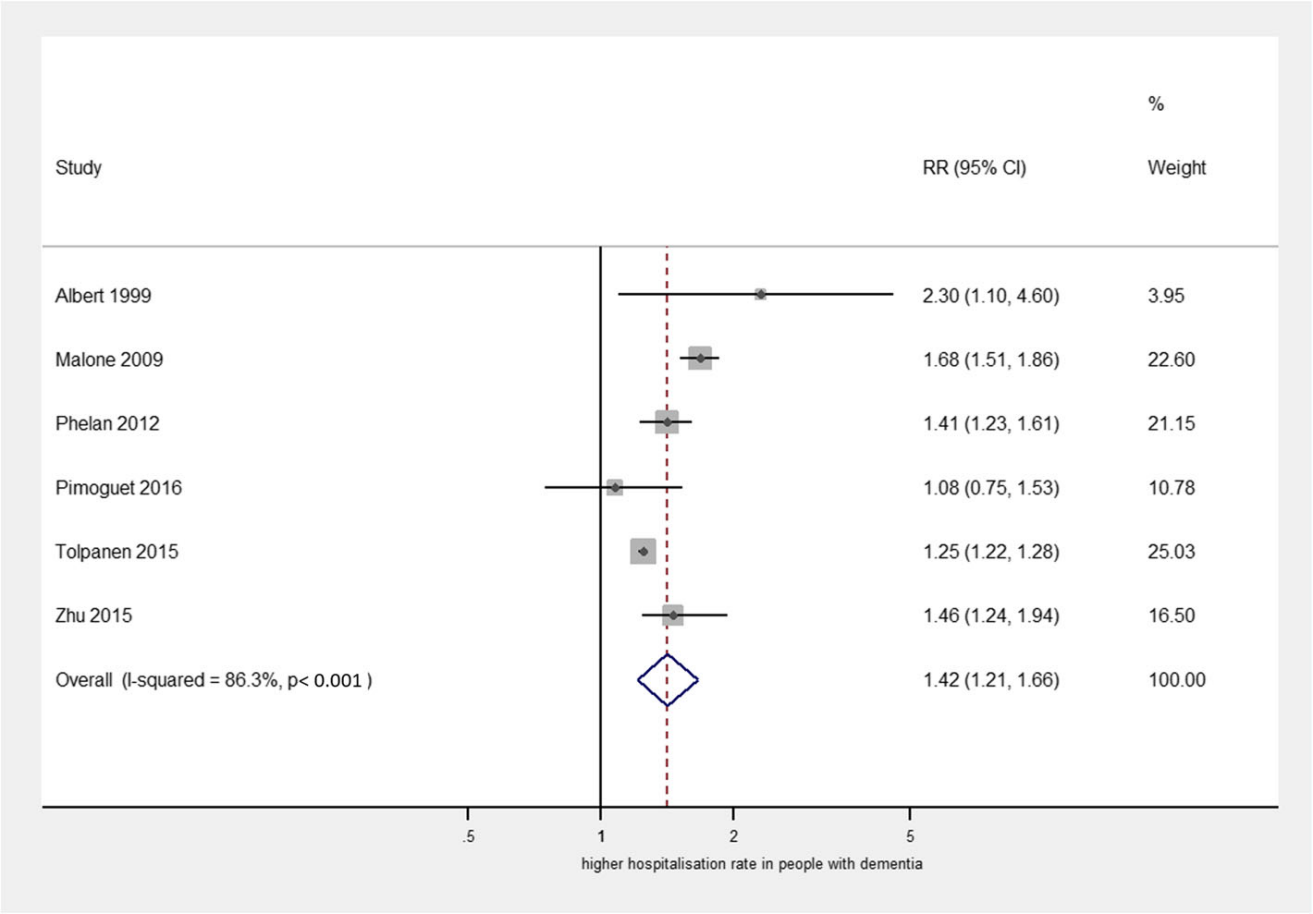
Forest plot of studies examining relative risk of hospitalisation for people with dementia compared to those without dementia, adjusted for age, sex, and physical comorbidity. Note: weights are derived from random effects meta-analysis

Six studies examined the risk of hospital admission in people with dementia compared to people without dementia, adjusting for at least age, sex, and physical comorbidity. Relative risk (RR) estimates in these papers ranged from 1.08 to 2.3. Figure 2 presents pooled relative risk estimate for hospitalisation in people with dementia compared to those without (RR = 1.42 (95% CI 1.21, 1.66), *p* < 0.001, *I*^2^ = 86.3%). There was a consistent direction of effect, and estimates were of similar magnitude. Begg’s test indicated a low risk of bias, so the overall strength of this evidence was graded as high (full data on the assignment of GRADE evidence strength is in Additional file 1: Table S4). Four other studies [23, 34, 41, 46] did not adjust for comorbidity and two of these found notably higher relative risk estimates (3.68 and 4.19), but we did not include these in our meta-analysis as we wished to examine the hospitalisation risk accounting for the potential confounding effect of physical illness; we were unable to include one other study which did not provide a specific *p* value or confidence interval [44].

Two studies examined hospitalisation risk for people with dementia at the end of life with one Taiwanese register study of people in the last year of life reporting RR for admission 1.14 (0.91–1.41) and the other study of Finnish people with dementia in the final 2 years of life showing lower admission risk (RR = 0.33 (0.31–0.35)). There was low confidence in the evidence as the studies are of moderate and low quality respectively, and they present contradictory estimates [47, 48].

### Rates of hospitalisation per year

Table 2 displays the rates of hospital admissions in people with dementia. The studies with the lowest risk of bias found the rate to be between 0.37/py and 1.26/py. Among studies that used research cohorts or clinical samples, the rate ranged from 0.16/py to 0.48/py, whereas in studies using national registers, the rate ranged from 0.23/py to 1.26/py. Where available, the percentage of participants hospitalised was also extracted (Additional file 1: Table S5), and in four studies at low risk of bias which followed participants for 1 year, 26–65% of study participants were admitted.

**Table 2:**
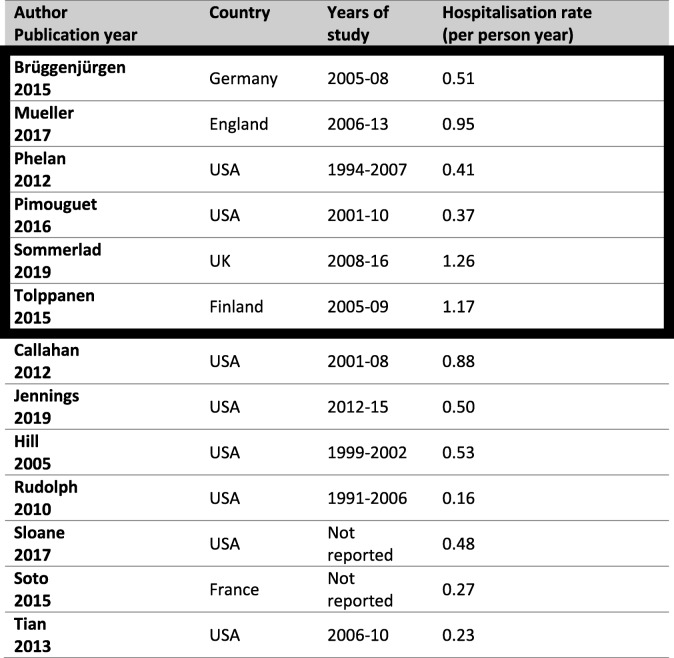
Hospitalisation rates of people with dementia

Bold outline indicates studies with lowest risk of bias

### Risk factors associated with hospitalisation

**Fig. 3:**
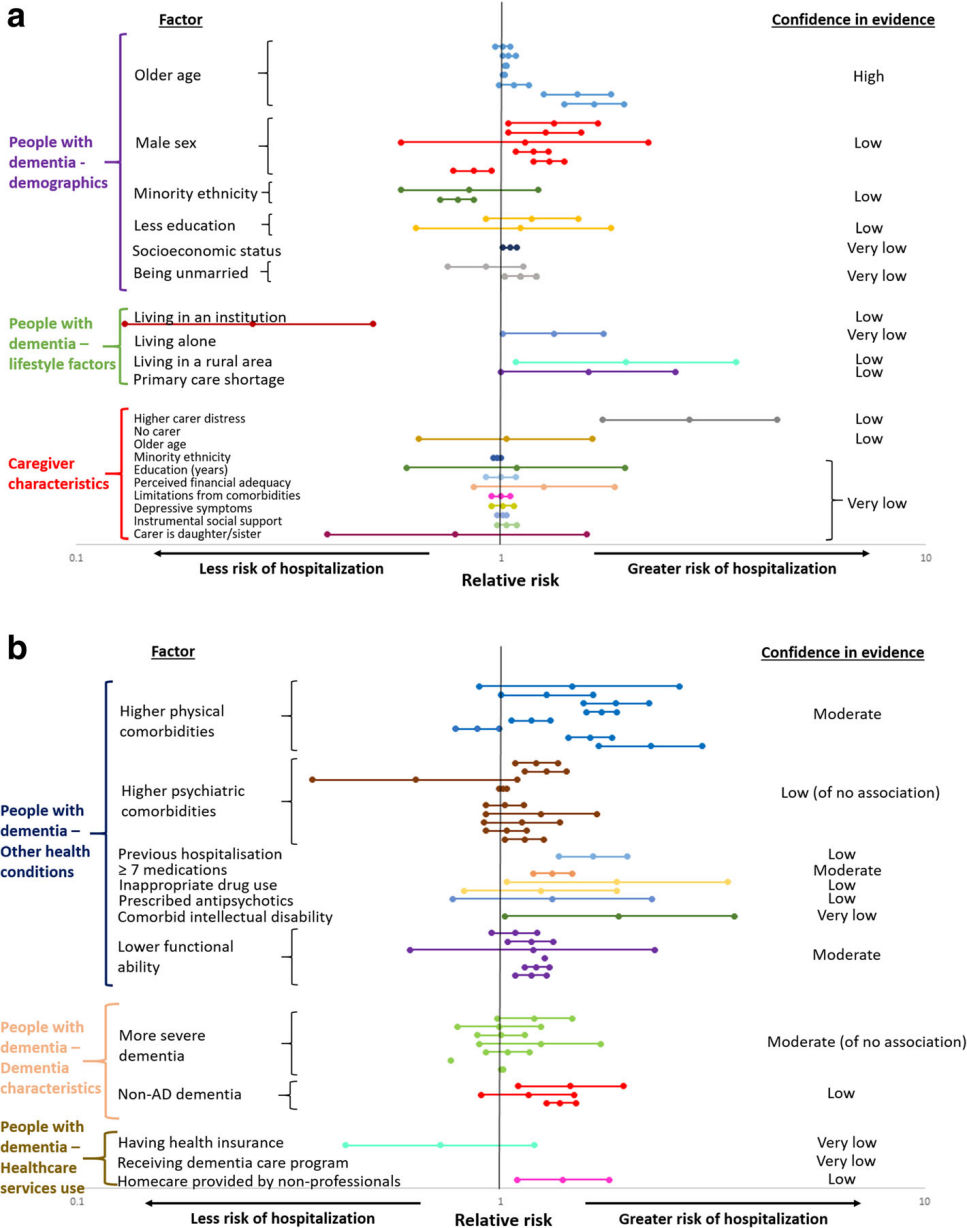
**a** Association of demographic characteristics of people with dementia or caregivers and hospitalisation: relative risk of admission and confidence in evidence. **b** Association of clinical characteristics of people with dementia and hospitalisation: relative risk of admission and confidence in evidence. Note: full information on risk factor classification and evidence grading is in Additional file 1: Table S6

Figure 3a and b show the associations between socio-demographic and clinical factors and hospitalisation in people with dementia, and each risk factor has been allocated a grade according to the confidence in the estimates. Full detail of the criteria used to assign GRADE levels of evidence strength for each risk factor is in Additional file 1: Table S6.

#### Factors relating to the person with dementia associated with admission

Older age was consistently associated with risk of hospitalisation across six studies; the odds ratio (OR) for hospitalisation with each additional year of age was OR = 1.02 in one study and OR = 1.04 in another [12, 30], and findings were consistent when considering people aged > 95 compared to < 75 (rate ratio = 1.66) [21]. After considering the quality of individual studies, overall consistency, and absence of evidence of publication bias (*p* = 0.60), we rated confidence in the effect of older age on increasing hospitalisation risk as high.

Having multimorbidities was associated with hospitalisation across six studies. A consistently large effect increased confidence in the finding, and there was no evidence of publication bias (*p* = 0.60). However, point estimates and confidence intervals around estimates varied widely, for instance, having other illnesses compared to none increased odds of hospitalisation hazard ratio (HR) = 1.28 in one study, OR = 1.47 in another, and HR = 1.87 in the third [37–39]. Therefore, confidence was graded as moderate.

There was moderate confidence in the association of lower level of functional ability with hospitalisation risk as four studies found a consistent direction of results with moderate magnitude (RR = 1.08 to 1.27), indicating that less independence contributed to a higher risk of hospitalisation. Similarly, the use of ≥ 7 (compared to 0–3) medications was significantly associated with a higher risk of admission in one high-quality (i.e. low risk of bias) study (HR = 1.32), and there was evidence of a dose-response effect [34], resulting in moderate confidence. There was also moderate confidence in the finding that severity of dementia was not associated as three studies at low risk of bias consistently found no effect of dementia severity on hospitalisation [37, 38, 51].

#### Factors relating to the primary caregiver associated with admission

There was low or very low confidence in all factors relating to the caregiver as only one study examined each association.

## Discussion

This is the first comprehensive systematic review of the rates and risk factors for hospitalisation in people with dementia. We found with high confidence an increased rate of hospitalisation of people with dementia compared to people without dementia. The pooled relative risk of hospitalisation for people with dementia compared to those without was 1.42 (95% CI 1.21–1.66) in studies adjusted for age, sex, and physical comorbidity. The increased risk of hospitalisation of people with dementia, in moderate or high-quality studies, ranged from 1.08 to 2.30. This wide range suggests that hospitalisation risk may be modified by differing healthcare provision. High-quality studies found general hospital admission rates for people with dementia to be between 0.37 and 1.26/py. There is strong evidence that older age is associated with admission and moderate evidence that presence of physical comorbidities, having a lower functional level and taking ≥7 medications, is associated with admission, while there was strong evidence that dementia severity alone is not associated with hospitalisation.

The organisation of health and social systems in different countries may affect hospitalisation risk, or differences in the relative risk for those with and without dementia could be due to the adjustments in analyses. One study that did not adjust for physical comorbidities found a risk of OR = 3.68 for people with dementia compared to those without, suggesting that worse physical illness explains part of the increased hospitalisation risk of people with dementia [52]. This study also analysed the hospitalisation risk according to the numbers of chronic conditions and found that dementia increased the risk of admission whether or not people had other long-term illnesses, no other long-term conditions (RR = 5.92, 5.60–6.27), or five (RR = 2.87, 2.71–3.04). The risk did not increase with each additional comorbid condition. Evidence for the association of dementia and hospitalisation risk at the end of life was low strength. People with dementia near end of life are usually very ill with multiple health problems, but families caring for someone with dementia at the end of life may wish to avoid hospitalisation because of the distress it can cause, which could explain varying results.

The variation in findings on hospitalisation rates (0.37 to 1.17/py) could be partly explained by the nature of the participant cohorts. Research cohort participants are often healthier than non-participants [53] and are subject to attrition of study participants; death is a common cause of attrition in studies of older populations [54]. Cohort studies may therefore become less representative over time and selectively include healthier participants. Lower hospitalisation rates could also be due to the method of outcome ascertainment, for example, asking family carers and patients about past admissions risks recall bias, whereas extracting data from a national database or register is likely to be more complete. Therefore, the highest rate of 1.17 admissions/py [43] may be the most reliable due to the large sample size and outcome derivation from a national register. Areas with more older people have lower emergency admission rates among older people [55] suggesting services are more integrated and prioritise community-based and ambulatory healthcare for older populations in these areas. Alternatively, there could be higher thresholds for emergency admissions due to the larger number of older people.

There is high confidence in the effect of older age, a risk factor which is unmodifiable. People with multimorbidities are unsurprisingly at higher risk of admission, but in one study, multimorbidity was associated with a higher risk of conditions judged as potentially manageable within primary care [12]. An earlier systematic review of reasons for admissions for people with dementia found that they are more likely to have been admitted to hospital for falls, fractures, and respiratory and urological infections than inpatients without dementia [56], so falls prevention and early recognition and management of respiratory and urinary tract infections and delirium may be valuable future intervention strategies. People with dementia often take many medications to manage existing comorbidities, and our study found moderate evidence that taking ≥ 7 medications increases the risk of hospitalisation. The study examining polypharmacy adjusted for patient comorbidities, suggesting that medication use may contribute to elevated hospitalisation risk independent of multimorbidity; potentially inappropriate prescribing is common in older people without dementia and associated with a higher risk of hospitalisation [57]. Polypharmacy is a potentially modifiable risk factor, and while this may partially reflect the severity or number of illnesses a patient has, it may be that some of the drugs individually or when given together are causing more harm than good. It may be that medication is reviewed less in people with dementia, and they remain on medications when no longer needed. Drug interactions are more likely with each drug taken [58] and are more likely to be harmful in older patients [59]. Non-adherence to medications may mean that medication is judged to be ineffective and more is added, or a person with dementia may accidentally take too much medication; both could increase the risk of harmful drug interaction in this population. A recent randomised controlled trial indicated that community-based deprescribing can be undertaken safely in older people without dementia, so it should be considered whether this could safely extend to people with dementia [60].

Having a lower functional ability was also associated with hospitalisation. Functional ability refers to basic tasks such as washing, and more intricate tasks such as cooking. In people with dementia, it can be difficult to ascertain whether loss of functionality is due to a combination of comorbidities, side effects of medication, or dementia. For instance, decreased ability to prepare food in people with dementia could lead to dangerous levels of malnutrition, dehydration, or weight loss [61], and therefore acute hospitalisation. Further loss of function could lead to an increase in the level of care needed to keep someone in the community and a decrease in the ability to seek and use appropriate help. Regarding dementia severity, we had moderate confidence that this was not associated, suggesting that it is a combination of physical and cognitive impairment and lack of external support, which increases admission risk, rather than cognitive impairment alone.

In addition to the risk posed by each individual risk factor, factors may interact with each other. It might be expected that people with dementia and at least one other comorbid condition are older than those with no comorbidities, take medication for their illnesses, and have a lower functional ability, therefore increasing their hospitalisation risk. Other studies have found that polypharmacy alone is associated with detrimental effects on functioning [62], which could further increase the risk of hospitalisation in this population. Rudolph et al. [37] discuss the theory of a multiplicative effect of risk factors and suggest patients with more than one risk factor comprise a high-risk group should be prioritised as recipients of future interventions to reduce hospitalisation.

### Strengths and limitations

The comprehensive data search and thorough methodology of this review avoids subjectivity of data extraction and limits the risk of significant evidence being missed. During the initial screening process, a sample of eligible papers was reviewed independently by a second researcher to ensure the robustness of the application of inclusion criteria. Similarly, the risk of bias was reviewed independently by a second researcher, and grades of evidence were based on research team consensus using the gold standard Cochrane approach. Our use of the PRISMA checklist ensured standardised reporting [18].

This review has limitations which mainly relate to the limitations of included studies. We were only able to examine papers from some countries, and different patterns of admission may exist elsewhere in the world. Studies of solely care home populations were excluded; therefore, the results are not generalizable to those living in long-term care and may only be applicable to community-dwellers. However, as two thirds of people with dementia live in the community [9], the results have the potential to inform care of a large proportion of the people currently living with dementia. In addition, we did not include studies whose population consisted of hospitalised patients, as they were already likely to be more unwell than those in the community. We therefore expected that re-admission rates would be higher in this patient group and risk factors for re-admission may differ from those for the first admission, and therefore not apply to the wider community-dwelling population. However, some risk factors for readmission reported in other studies may apply to the first hospitalisation. When extracting the results, we focussed on “all-cause” hospitalisation and used the definition of hospitalisation provided by each study and studies may have defined this differently. Similarly, we used data for “avoidable” hospitalisation when all-cause hospitalisation data was not provided, and these definitions differed between studies. Avoidable admissions were termed “primary care sensitive” by Pimouguet, “ambulatory care sensitive” by Thorpe, “preventable” by Bynum, and “unplanned” or emergency by Mueller and Sommerlad [11, 12, 34, 41, 52]. Exactly which conditions fall into these categories varies, although there is a consensus that it comprises conditions which could be treated in the home or by primary care. It may be that only a rigorous randomised controlled trial with a well-designed intervention can determine the extent to which admissions are avoidable. Potential risk factors were classified in different ways between included studies, which prohibited combining estimates using meta-analysis. Finally, all non-randomised studies are susceptible to selection bias and residual confounding, so causality cannot be proven from observational studies.

## Conclusions

This review finds hospital admission rates among people with dementia to be between 0.37/py and 1.17/py and strong evidence that people with dementia have 1.42 times higher risk of hospitalisation compared to people without dementia. Our evidence on admission rates of people with dementia informs policymakers aiming to ensure appropriate current and future provision of hospital care for people with dementia. Our study identifies people with dementia at high risk of hospitalisation—those who are older with physical comorbidities—and suggests that reducing polypharmacy and ameliorating functional impairment potentially will reduce admission risk. This informs clinicians treating people with dementia and future interventions aiming to reduce hospitalisation of people with dementia. There are indications of other potentially modifiable hospitalisation risk factors, but we were unable to make confident conclusions so rated them as low or very low due to the lack of evidence; these may warrant further detailed research. Identifying modifiable risk factors for hospital admission and devising effective approaches to address these have huge potential to improve the lives of people affected by dementia.

## Additional files

Additional file 1: **Table S1.** Full list of search terms. **Table S2.** Full data extracted from included studies and full references. **Table S3.** Quality rating criteria and scores for included studies. **Table S4.** Risk of hospitalisation in people with dementia compared to people without dementia: full details of GRADE rating of evidence strength. **Table S5.** Percentage of study participants hospitalised in the study period. **Table S6.** Association of potential risk factors with hospitalisation in people with dementia: full details of GRADE rating of evidence strength for risk factors. (DOCX 147 kb)

Additional file 2: Study protocol. (PDF 100 kb)

## Data Availability

All data generated or analysed during this study are included in the published articles and its additional files.

## Abbreviations

ACSC: Ambulatory care-sensitive conditions
CI: Confidence interval
GRADE: Grading of Recommendations, Assessment, Development and Evaluation
HR: Hazard ratio
NOS: Newcastle-Ottawa Scale
OR: Odds ratio
Py: Person-years
RR: Relative risk
