# Supplementary material for: Hospitalisation rates and predictors in people with dementia: a systematic review and meta-analysis
Source: BMC Med. 2019 Jul 15;17:130. doi: 10.1186/s12916-019-1369-7 (PMC6628507; doi:10.1186/s12916-019-1369-7)
Supplement: Supplementary file 1 — Table S1. Full list of search terms. Table S2. Full data extracted from included studies and full references. Table S3. Quality rating criteria and scores for included studies. Table S4. Risk of hospitalisation in people with dementia compared to people without dementia: full details of GRADE rating of evidence strength. Table S5. Percentage of study participants hospitalised in the study period. Table S6. Association of potential risk factors with hospitalisation in people with dementia: full details of GRADE rating of evidence strength for risk factors. (DOCX 147 kb) [file 12916_2019_1369_MOESM1_ESM.docx]

## Table S1: Full list of search terms

**Table S1a: search terms for Ovid MEDLINE (Epub Ahead of Print, In-Process & Other Non-Ovid MEDLINE Epub Ahead of Print, In-Process & Other Non-Indexed Citations, Ovid MEDLINE Daily and Ovid MEDLINE 1946 to 22 October 2018)**

| **PIECOS criteria** | **Search Terms** | **Number of papers found** |
| --- | --- | --- |
| **Population** | n/a | - |
| **Exposure** | 1 exp Dementia/  2 dementia.mp.  3 Alzheimer* Disease.mp.  4 Creutzfeldt Jakob.mp  5 vascular dementia.mp.  6 neurofibrillary tangles.mp.  7 frontotemporal lobar degeneration.mp.  8 frontotemporal dementia.mp.  9 kluver-bucy.mp.  10 lewy bod*.mp.  11 multi infarct dementia.mp.  12 primary progressive aphasia.mp.  13 presenile dementia.mp.  14 senile dementia.mp. | 144651  107722  129365  7814  5746  8342  2401  5760  257  8546  900  953  573  2793 |
|  | 15 1 or 2 or 3 or 4 or 5 or 6 or 7 or 8 or 9 or 10 or 11 or 12 or 13 or 14 | 218499 |
| **Comparator** | n/a | - |
| **Outcome** | 16 exp Hospitalization/  17 hospitali#ation.mp.  18 hospital stay.mp.  19 hospital admission.mp.  20 Hospital care.mp.  21 length of stay.mp.  22 patient admission.mp.  23 patient discharge.mp.  24 patient readmission.mp. | 205748  180230  64047  20485  8941  99371  22519  26077  13144 |
|  | 25 16 or 17 or 18 or 19 or 20 or 21 or 22 or 23 or 24 | 351999 |
| **Study Design** | 26 exp case-control studies/  27 exp Longitudinal Studies/  28 exp Cohort Studies/  29 exp epidemiologic studies/  30 exp Cross-Sectional Studies/  31 case control stud*.mp.  32 longitudinal stud*.mp.  33 Cohort stud*.mp.  34 follow-up stud*.mp.  35 cross-sectional stud*.mp.  36 epidemiolog* stud*.mp. | 909745  114681  1732640  2146587  263225  276174  146116  315913  608854  304872  83416 |
|  | 37 26 or 27 or 28 or 29 or 30 or 31 or 32 or 33 or 34 or 35 or 36 | 2341738 |
| **AND** | 38 15 and 25 | 3807 |
| **AND** | 39 38 and 37 | 1632 |
| **LIMIT** | 40 Limit 39 to humans | 1571 |
| - .mp. = title, abstract, original title, name of substance word, subject heading word, keyword, heading word, protocol supplementary concept word, rare disease supplementary concept word, unique identifier, synonyms - / = Subject heading - exp = explode | | |

**Table S1b: search terms for Embase Classic+Embase 1947 to 22 October 2018**

| **PIECOS criteria** | **Search Terms** | **Number of papers found** |
| --- | --- | --- |
| **Population** | n/a | - |
| **Exposure** | 1 *dementia/  2 dementia.mp.  3 alzheimer* disease.mp.  4 Creutzfeldt Jakob.mp.  5 vascular dementia.mp.  6 frontotemporal lobar degeneration.mp.  7 frontotemporal dementia.mp.  8 lewy bod*.mp.  9 multi infarct dementia.mp.  10 primary progressive aphasia.mp.  11 presenile dementia.mp.  12 senile dementia.mp. | 50570  174204  204737  12357  8977  3580  14712  15643  1075  2125  2564  5313 |
|  | 13 1 or 2 or 3 or 4 or 5 or 6 or 7 or 8 or 9 or 10 or 11 or 12 | 326125 |
| **Comparator** | n/a | - |
| **Outcome** | 14 *hospitalization/  15 *hospital admission/  16 *hospital patient/  17 hospitali#ation.mp.  18 hospital stay.mp.  19 hospital admission.mp.  20 hospital care.mp.  21 length of stay.mp.  22 patient admission.mp.  23 patient readmission.mp. | 31023  16003  21501  391483  105000  173657  28732  159244  1422  539 |
|  | 24 14 or 15 or 16 or 17 or 18 or 19 or 20 or 21 or 22 or 23 | 688115 |
| **Study Design** | 25 exp case-control study/  26 exp longitudinal study/  27 exp cohort analysis/  28 exp epidemiology/  29 exp Cross-Sectional Study/  30 case control stud*.mp.  31 longitudinal stud*.mp.  32 Cohort stud*.mp.  33 follow up stud*.mp.  34 cross-sectional stud*.mp.  35 epidemiolog* stud*.mp. | 142384  112223  364455  2967305  249862  179709  143865  224878  64878  292125  106311 |
|  | 36 25 or 26 or 27 or 28 or 29 or 30 or 31 or 32 or 33 or 34 or 35 | 3552465 |
| **AND** | 37 13 and 24 | 8440 |
| **AND** | 38 36 and 37 | 4010 |
| **LIMIT** | 39 Limit 38 to human | 3876 |
| - .mp. = title, abstract, heading word, drug trade name, original title, device manufacturer, drug manufacturer, device trade name, keyword, floating subheading word - / = Subject Heading - exp = explode - = focus | | |

**Table S1c: search terms for PsycINFO 1806 to 22 October 2018**

| **PIECOS criteria** | **Search Terms** | **Number of papers found** |
| --- | --- | --- |
| **Population** | n/a | - |
| **Exposure** | 1 exp dementia/  2 dementia.mp.  3 alzheimer* disease.mp.  4 Creutzfeldt Jakob.mp.  5 vascular dementia.mp.  6 neurofibrillary tangles.mp.  7 Frontotemporal lobar degeneration.mp  8 frontotemporal dementia.mp.  9 kluver bucy.mp.  10 lewy bod*.mp.  11 multi infarct dementia.mp.  12 primary progressive aphasia.mp.  13 presenile dementia.mp.  14 senile dementia.mp. | 69103  63967  54603  969  3611  2121  1261  3337  122  3481  632  759  432  2086 |
|  | 15 1 or 2 or 3 or 4 or 5 or 6 or 7 or 8 or 9 or 10 or 11 or 12 or 13 or 14 | 94675 |
| **Comparator** | n/a | - |
| **Outcome** | 16 hospitali#ation.mp.  17 exp Hospitalization/  18 hospital stay.mp.  19 length of stay.mp.  20 patient admission.mp.  21 patient discharge.mp.  22 patient readmission.mp.  23 exp Hospital Admission/  24 exp Hospitalized Patients/  25 hospital admission.mp.  26 hospital care.mp. | 29691  21250  2579  4731  124  224  53  4833  12151  6151  1813 |
|  | 27 16 or 17 or 18 or 19 or 20 or 21 or 22 or 23 or 24 or 25 or 26 | 52907 |
| **Study Design** | 28 exp longitudinal studies/  29 exp cohort analysis/  30 exp epidemiology/  31 longitudinal stud*.mp.  32 case control.mp  33 cohort stud*.mp.  34 follow up stud*.mp.  35 cross-sectional stud*.mp.  36 epidemiolog* stud*.mp. | 15921  1250  46743  54380  9687  17748  12228  21421  30009 |
|  | 37 28 or 29 or 30 or 31 or 32 or 33 or 34 or 35 or 36 | 176442 |
| **AND** | 38 15 and 27 | 1684 |
| **AND** | 39 38 and 37 | 303 |
| **LIMIT** | 40 Limit 39 to “Human” | 297 |
| - mp. = title, abstract, heading word, table of contents, key concepts, original title, tests & measures - / = Subject Heading - exp = explode | | |

## Table S2: Full data extracted from included studies

| **Author**  **Year**  **Country**  **Cohort details** | **Total number of participants (number with dementia if different)**  **% follow up (if available)** | **Population description (mean age and sex)** | **Average severity of dementia (if available)** | **Method of diagnosis** | **Length of study**  **(average follow up time of people with dementia)** | **Primary study outcomes** | **Method of outcome ascertainment** | **Adjusted risk of hospital admissions VS people without dementia**  **(OR, 95% confidence interval)** | **Rate of hospital admission (per person year) or**  **% of cohort hospitalised** | **Factors associated with hospital admission (adjusted for all covariates taken at baseline)** | **Covariates taken at baseline** |
| --- | --- | --- | --- | --- | --- | --- | --- | --- | --- | --- | --- |
| **Albert**  **1999** [1]  USA  Research cohort (WHICAP) | 2,003  (400) | Female: 71%  <70: 22%  71-74: 26% 75-79: 14%  80-84: 11%  >85: 7% | No information | Standardized assessment and examination | 1996 - 1997  (21 months) | Number of admissions, length of stay, discharge diagnoses | Hospital records; carer and self-report | Mild-moderate: 1.20 (0.7-1.8)  Severe: 2.30 (1.1-4.6)  No AD (Ref) | 70 (17.5%) in 1.75 years | N/A | Sex, Age, Residence (Community or Nursing home), ethnicity, Education, Comorbidity Index, AD diagnosis, death in Reporting Period |
| **Aupperle**  **2000** [2]  USA  Clinical sample | 58  72.5% | 81.6 | No information | Standardized assessment and examination | No information  (1 year) | Usage of health services | Carer report | N/A | 16 (27.5%) | Type of physician  Primary care physician plus geriatric psychiatrist (ref)  Primary care only  p=<0.05 | Age, sex, marital status, living arrangement, relationship of caregiver, assessment of physical impairment, Clinical Dementia Rating, caregiver burden, prescription of donepezil, utilization of health services |
| **Axmon**  **2016** [3]  Sweden  Swedish National Patient registry | 15,872  (271) | ID no dementia:  Age: 53  Female: 45%  N=7,720  ID and dementia:  Age: 61 Female: 55%  N=199  No ID no dementia:  Age: 53  Female: 45%  N=7,855  No ID and dementia:  Age: 71  Female: 50%  N=72 | No information | No detail of assessment or examination. | 2002-12  (11 years) | Number of planned and unplanned in- and out-patient visits, length of stay | Hospital records | 4.19  (1.88–9.32) | 32 (44.0%) in 1 year | Having intellectual disability  Planned admissions:  0.74 (0.33–1.58)  Unplanned admissions: 1.90 (1.02–3.55) | Age at end of study, sex, time in study |
| **Browne**  **2017** [4]  UK  National register (CPRD) | 4,999  67.7% | 81.4  Female: 65.0% | No information | Two experienced clinicians reviewed diagnoses, with disagreements settled through discussion with a further two clinicians. | 2.97 years | Counts of primary care  consultations, hospitalisations, numbers of medicines prescribed, time-to-death | Hospital records | N/A | N/A | **Age** <75 (ref)  75–79  1.32 (1.15-1.50)  80–84  1.28 (1.13-1.46)  85–89  1.55 (1.36-1.76)  ≥90 years  1.66 (1.41-1.95)  **Comorbidities** 0-1  0.88 (0.78-0.99)  2-3 (ref)  4-5  1.19 (1.07-1.32)  ≥6  1.62 (1.44-1.83) | Age, sex, socioeconomic  status, primary care practice, dementia medications (AchEI and memantine) |
| **Brüggenjürgen**  **2015** [5]  Germany  Insurance register (DAK-Gesundheit) | 20,000  100% | 2005  79 Female: 72%  N=18,745  2006  80.1  Female: 73%  N=18,945  2007  80.4 Female: 72%  N=20,484  2008  80.7 Female 73%  N=20,903 | No information | No detail of assessment or examination. | 2005-2008  (4 years) | Number of hospitalisations, inpatient days, ambulatory physician visits, number of drugs used, total number of defined daily doses of prescribed drugs, costs | Hospital records | N/A | 0.51 | N/A | Age, sex |
| **Bynum**  **2004** [6]  USA  Insurance register (Medicare) | 1,238,895  (103,512) | 81.9  Female: 68% | No information | No detail of assessment or examination. | 1999  (1 year) | Expenditures, rate of all cause hospitalisation, rate of ACSC admissions | Hospital records | 3.68 (3.62–3.73) | N/A | N/A | Age, sex, ethnicity, comorbidity index, occurrence of death within the year |
| **Callahan**  **2012** [7]  USA  Insurance registers (Medicare, MDS, OASIS, Medicaid) | 4,197 | 71.9  Female 68.8% | No information | No detail of assessment or examination. | 2001 – 2008  (5.2 years) | Time to death, nursing home admission, comorbidities, transitions in care | Hospital records | N/A | 0.88 | N/A | Age, sex, ethnicity, death, comorbid conditions |
| **Chen**  **2014** [8]  UK  National registers (CPRD) | 11,688  (3,896) | 79.9  Female: 65% | No information | No information | 2008 – 2010  (2 years) | Consultation rate, specialist referral rate, hospital admission, LOS | Hospital records | GOA cohort: 18.4% of 3,407 GOAs (613.26)  AD vs GOA P=<0 .0001 | 415.9 (23.3%) | N/A | Age, sex, region (England, Northern Ireland, Scotland, Wales), baseline rates of selected comorbidities, baseline Comorbidity Index score. |
| **Chen**  **2017** [9]  Taiwan  Insurance registers (Taiwan National Health Insurance Research Database) | 2,724  (908)  100% | Male: 522 (57.49%) | No information | No detail of assessment or examination. | 2002-2011  (9 years) | Utilization of healthcare during the last year of life: hospital admissions, length of inpatient stays, emergency department (ED) visits, non-palliative interventions, comorbidities. | Hospital records | 1.14 (0.91–1.41) | 748 (82.3%) in the last year of life | N/A | Mean age at death, age at death, sex, comorbidities |
| **Cortes**  **2008** [10]  France  Research cohort (REAL-FR) | 686  59% | 77.86  Male: 198 (29.86%) | Mean MMSE: 20.01  Mean ADAS-cog: 17.85 | No detail of assessment or examination. | No information  (2 years) | Institutionalization, death; hospitalisation; evolution toward severe dementia, loss of independence, NPI total score, nutrition status, rates of stopping or changing medication | Carer and self-report | N/A | 91 (26.1%) in 1 year | N/A | Age, sex, duration of AD, living arrangement (with relatives/alone), specific AD treatment (y/n), MMSE, ADL, ADAS-Cog, CDR, NPI, MNA |
| **Forma**  **2011** [11]  Finland  National registers (Care Register for Health Care, Care Register for Social Welfare and Home Care Census) | 145 944  (34,232)  100% | With dementia  85.0  Female: 69.6% | No information | No detail of assessment or examination. | 1998-2003 (2 years) | Hospital inpatient care, long-term care, regular home care | Hospital records | 0.33 (0.31-0.35) | 22,011 (64.3%) in the last 2 years of life | University hospital care  15.1% vs 29.5% (p=<0.001)  General hospital care 38.3% vs 59.1% (p=<0.001) | Age, sex, dementia (Y/N), year of death, sum of other comorbidities |
| **Hill**  **2005** [12]  USA  Insurance register (Medicare) | 14,023  (3,357)  100% | VaD  79.5  Female: 55%  N=678  AD  79.4  Female: 62%  N=1,722  Other dementia  80.9  Female: 66%  N=957 | No information | No detail of assessment or examination. | 1999-2002  (3 years) | Healthcare utilization and costs for VaD compared with cardiovascular disease (CVD) patients | Hospital records | N/A | 0.59 | N/A | N/A |
| **Jennings**  **2019** [13]  USA  Insurance register (Medicare) | 3,249  100% | All-cause dementia  82.2 years  Female: 64.9% | Mean disease duration 3years | No detail of assessment or examination | 2012-2015  (1 year for assessment of hospitalisation rate.  1.75y for effect of dementia care program) | Hospitalisations, Medicare costs, emergency department visits, readmissions | Insurance register | N/A | 0.50 admissions/yr | Comprehensive dementia care program:  Difference-in-Differences Estimate for Utilization = −1 (−13 to 11) | Age, sex, race, ethnicity, hierarchical Condition Category score, Alzheimer disease diagnosis and number of days of fee-for-service coverage in quarter |
| **Kedia**  **2017** [14]  USA  Insurance register (Medicare) | 96,124  (8,533)  100% | Coexisting dementia and cancer  N=1,294  Female: 691 (53.40%)  Dementia only  N=8,533  Female: 6,233 (73.05%) | No information | No detail of assessment or examination. | 2009  (1 year) | General hospitalisation, readmissions within 30 days, length of stay, ICU use, discharge status and destinations, psychiatric hospitalisation, ED visits, physician visits, nursing home stays, hospice stays | Hospital records | 3.70 (3.50-3.90) | 2,220 (25.96%) in 1 year | N/A | Age, ethnicity, sex, state subsidy status, medical comorbidities and location |
| **Kunik**  **2003** [15]  USA  Research Cohort (Houston Veteran Affairs Clinic) | 864  With BPSD: n=567 (66%)  No BPSD:  n=297 (34%) | 71  Female: 45 (5%) | No information | No detail of assessment or examination. | 1997-1999  (22 months) | Inpatient bed days, number of outpatient clinic visits | Hospital records | N/A | 356 (41.2%) in 1.8 years | Dementia no BPSD (ref)  Dementia with BPSD 1.56 (1.10-2.23) | Days in study, age, sex, ethnicity, marital status, medical comorbidity, psychiatric comorbidity |
| **Malone**  **2009** [16]  USA  National register (HealthCore) | 5,396  100% | 76.9  Female: 67.7% | No information | No detail of assessment or examination | 2000-2006 | Resource utilization | Hospital records | 1.68 (1.51-1.86) | 1338 (24.8%) in 5 years | Higher age  1.02 (1.01-1.02)  Sex  0.86 (0.77-0.95) | Age, sex, comorbidities |
| **Maust**  **2017** [17]  USA  Research cohort (ADAMS) | 332  With dementia and BPSD: n=58  Dementia and no BPSD: n=274 | With dementia and BPSD:  Age: 83.5  Female: 77.0%  Dementia and no BPSD:  Age: 84.7  Female: 67.5% | With BPSD  Mean MMSE: 11.7  No BPSD  Mean MMSE: 17.9 | Standardized assessment and examination | 2002-2008 | Presence of BPSD, Medicare expenditure, number of ED visits, acute inpatient hospitalisations | Hospital records | N/A | 217 (65.2%) in 1 year | Informant distress related to BPSD  Dementia no BPSD (ref)  Dementia with BPSD  0.63 (0.36-1.09) p=0.09 | Age, sex, ethnicity, education, informant co-residency, nursing home residency, dementia severity, comorbidities |
| **Miu**  **2018** [18]  Hong Kong  Clinical sample | 267 | 79.9  Female: 68.8% | Mean MMSE: 17 | No detail of assessment or examination | 2013-2015 | Frequency of hospitalisations, principal diagnosis for admission, risk factors for admission | Hospital records | N/A | 36.7% in 1 year | Age  1.11 (1.06-1.17)  CCI  1.34 (1.08-1.67) | Age, sex, MMSE, mBI, dementia subtype |
| **Mueller**  **2017** [19]  UK  Clinical register (CRIS) | 970 | DLB cohort  Mean age at diagnosis: 79.9  Female: 50%  AD cohort  Mean age at diagnosis: 80.3  Female: 50% | DLB cohort  Mean MMSE at diagnosis: 19.2  AD cohort  Mean MMSE at diagnosis: 18.7 | A subset of the sample double checked by clinicians with experience of diagnosing DLB | 2006-2013  DLB cohort mean follow up time: 10.1 months  AD cohort mean follow up time: 11.3 months | Planned and unplanned general hospitalisation | Hospital records | DLB vs General Population  Admission ratio 1.22 (1.06-1.39)  AD vs General Population  Admission ratio 0.91 (0.84-0.99) | 0.95  435 (46.3%) in 0.9 years | Dementia with Lewy Bodies  All admissions  1.07 (0.85–1.35) p=0.54  Planned admissions  1.10 (0.70–1.72) p=0.67  Unplanned admissions  1.06 (0.80–1.39) p=0.68 | Age, sex, MMSE score at diagnosis, comorbidities, hospitalisation in year before dementia diagnosis, ethnicity, marital status, deprivation score, mental health and functional problems score |
| **Mueller**  **2018** [20]  UK  Clinical register (CRIS) | 4,668 | Female: 64.2% | MMSE: 18.4 | No information | 2011-2013  (2 years) | Unplanned hospitalisations, ED attendance, death | Hospital records | N/A | 3,105 (66.5%) in 2 years | Taking 7+ medications  0-3 medications (ref)  4-6 medications  1.12 (1.02–1.22) p=<0.05  7+ medications  1.32 (1.19–1.47) p=<0.01 | Age, sex, marital status, ethnicity, deprivation score, MMSE score, comorbidities, mental health problems at time of diagnosis, hospitalisation in the two years prior to dementia diagnosis, dementia subtype |
| **Nourhashemi**  **2005** [21]  France  Research cohort (REAL-FR) | 677  84.6% | Living alone: 186 79.4  Male: 6.46%  Living with others: 491  76.9  Male: 38.28% | Living alone  Mean MMSE: 19.8  Living with others  Mean MMSE: 20.1 | Standardized assessment and examination | No information  (1 year) | MMSE, NPI, MNA, ADL, carer burden, medical and non-medical care utilization | Carer and self-report | N/A | 154 (26.8%) in 1 year | % hospitalised:  Living with others (27.4%)  Living alone (25.8%)  No significant differences | Age, sex, home services (health services, other services), MMSE, ADL, NPI, MNA, carer burden |
| **Phelan**  **2012** [22]  USA  Research cohort (ACT) | 3,019  (494)  95% | 75.3 | Those who developed dementia: 90.3  Never developed dementia: 93.6  (0 worst, 100 best) | Standardized assessment and examination | 1993-2007  (9.6 years) | Rate of hospitalisations | Hospital records | 1.41 (1.23-1.61) | 0.41 | N/A | Age, sex, ethnicity, education, living alone, calendar year of admission, self-rating of health, count of number of activities of daily living performed with difficulty |
| **Pimouguet**  **2016** [23]  Sweden  Research cohort (SNAC-K) | 3,000  (175)  77% | 82.0  Female: 73.7% | 26.9 | Standardized assessment and examination | 2001-4  (4.7 years) | Hospitalisation for primary care sensitive conditions | Hospital records | 2.30 (1.35-3.92) (complete cohort) | N/A | PCSC  Acute:  2.99 (1.29–6.92)  Chronic:  1.63 (0.86–3.11) | Age, sex, education, living arrangements, MMSE, ADL, IADL disability, living alone, chronic conditions, inappropriate drug use, healthcare assistance |
| **Rudolph**  **2010** [24]  USA  Research cohort (MADRC) | 827 | 75.8  Female: 480 (58%) | Mean: 1.9  (0-5 points, 5 worst) | Standardized assessment and examination | 1991 - 2006  3.0 years | Acute hospitalisation | Carer and self-report | N/A | 0.16  542 (66.0%) in 2.2 years | Age  75+  1.51 (1.26–1.81)  Sex  Male  1.27 (1.04–1.54)  Ethnicity  Non-white  0.84 (0.58–1.22)  Education  <12 years  1.18 (0.92–1.52)  Marital status  Unmarried  HR=0.92 (0.75–1.13)  family history of dementia (y/n)  1.15 (0.83–1.58)  BIMC score  >=15  0.99 (0.79–1.24)  Dementia Severity  >=2 points  1.20 (0.98–1.47)  Duration of symptoms before diagnosis  <=1.5 years  1.26 (1.02–1.56)  Speed of initial onset  Rapid  0.83 (0.56–1.22)  Slow (ref)  Course of deterioration  Fluctuating or stepwise  0.92 (0.55–1.54)  Stable or improving (ref)  Comorbidities  >=1  1.87 (1.57–2.23)  Previous acute hospitalisation  1.65 (1.37–1.99)  Adjusted for all listed covariates | Age, sex, ethnicity, education, marital status, family history of dementia, cognitive function, dementia severity, duration of symptoms before diagnosis, speed of initial onset, course of deterioration, comorbidities, previous acute hospitalisation |
| **Russ**  **2015** [25]  Scotland  Research cohort (SDRIR) | 730  95.8% | 76.3  Female: 47.8% | Mean MMSE score of group admitted to hospital: 21.3  Mean MMSE score of group not admitted to hospital: 21.3 | Standardized assessment and examination | No information  (1.2 years) | General hospital admission | Hospital records | N/A | 274 (37.5%) in 1.2 years | Age (per 5 years older)  1.07 (0.99-1.16)  AD vs all other dementia subtypes  0.86 (0.67–1.11)  Mixed dementia vs all others  0.86 (0.61–1.22)  VaD vs all others  1.21 (0.84–1.75)  Other (PDD, DLB, FTD, unspecified) vs all others  1.67 (1.09–2.56)  Vascular risk factors  1.03 (0.81–1.30)  Any comorbidity  1.28 (1.00–1.65)  Having a carer vs none  1.03 (0.64–1.64)  MMSE score  1.00 (0.88–1.14)  IADL score  1.08 (0.95–1.22)  PSMS score  1.18 (1.04–1.33)  Total NPI score  1.22 (1.09–1.37)  NPI caregiver distress score  1.14 (1.02–1.28)  Dementia severity  More advanced dementia  p-trend =0.45  CDR sum of values  1.04 (0.92–1.17)  Total NPI score=0  1.29 (0.95–1.76)  Total NPI score=  1-14  1.29 (0.95–1.76)  Total NPI score=15+  1.82 (1.30–2.56)  NPI category: agitation  Per SD increase 1.28 (1.14–1.43)  Potentially inappropriate medications  1.24 (0.82–1.88)  Prescribed AChEI  0.87 (0.67–1.14)  Prescribed antipsychotics  1.32 (0.77–2.27) | Age, sex |
| **Sköldunger**  **2015** [26]  Sweden  Research cohort (SNAC) | 4,108  (319)  99.4% | 74.8  Female: 2580 (62.8%) | No information | No detail of assessment or examination | 2001–2004  (3 years) | Risk of hospitalisation, death | Hospital records | N/A | N/A | Higher age  1.01 (0.97–1.05)  Sex  Female  0.87 (0.45–1.71)  Residential setting  Community (Ref)  0.26 (0.13–0.50)  Education  Elementary (Ref)  Additional:  0.90 (0.55–1.57)  ADL  Independent (Ref)  Dependent:  1.19 (0.61–2.30)  Co-morbidity  None (Ref)  Co-morbidities:  1.47 (0.89–2.63)  Inappropriate Drug Use  No (ref)  1.88 (1.03–3.43) | Age, sex, education level, residential setting, comorbidities, number of drugs, ADL score, inappropriate drug use |
| **Sloane**  **2017** [27]  USA  Clinical sample | 136  85% | 78  Female: 85 (63%) | Cognitive Impairment - CPS  Mild: 10 (7%)  Moderate: 82 (60%)  Severe: 44 (33%) | No detail of assessment of examination | No information  (5.7 months) | Symptoms of PWD, incidence of behavioural, organ-specific, and nonspecific medical symptoms, ED visits, hospitalisations, death | Carer and self-report | N/A | 0.48  31 (22.79%) in 6 months | N/A | Characteristics of the PWD  age, sex, time since diagnosis, cognitive impairment (CPS – mild, moderate, severe), physical health as rated by caregiver, need for assistance  Characteristics of the caregiver  relationship to PWD, lives with PWD, age, sex, education, employment, has some medical training, ethnicity, self-rated health |
| **Sommerlad**  **2019** [28]  USA  Clinical register (CRIS) | 10,137  100% | 82.1 years  Female = 61.8% | Mean MMSE: 18.6 | No detail of assessment of examination | 2008-16  2.5 years median f/u | Emergency and elective hospitalisation | Hospital records | Age/sex-adjusted IRR  Emergency admissions = 2.06 (1.95, 2.18)  Elective admissions = 1.00 (0.93, 1.07) | 75.9% admitted during 2.5y median follow-up  50.6% admitted during first year after diagnosis  Admission rate = 1.26/py  - Emergency = 0.90/py  - Elective = 0.35/py | Emergency admissions:  Age (per year increase)  IRR = 1.03 (1.02-1.03)  Female sex  0.77 (0.71-0.84)  Minority ethnicity  0.79 (0.72-0.86)  Unmarried  1.11 (1.02-1.21)  Higher socio-economic deprivation (per decile increase)  1.05 (1.01-1.09)  MMSE (per 1point decrease)  1.01 (1.00-1.01)  Agitated behaviour  1.02 (0.92-1.13)  Self-injury  1.24 (0.92-1.68)  Problem-drink/drugs  1.12 (0.91-1.38)  Physical illness  1.73 (1.59-1.88)  Hallucinations  1.03 (0.92-1.15)  Depressed mood  1.14 (1.02-1.26)  Daily living problems  1.18 (1.08-1.28)  Living condition problems  1.16 (1.04-1.30)  Non-Alzheimer’s dementia  1.38 (1.28-1.50)  Year of diagnosis (per 1y later)  1.03 (1.01-1.05) | Age, sex, ethnicity, marital status, socioeconomic deprivation, MMSE, agitated behaviour, self-injury, problem drink/drugs, physical illness, depressed mood, hallucinations, daily living problems, living condition problems, dementia subtype, year of diagnosis |
| **Soto**  **2015** [29]  France  Research cohort (PLASA) | 1,131 | Living alone: 348 (30.8%)  Age: 81.1  Female: 314 (90.2%)  Living with others: 783 (69.2%)  Age: 79.0  Female: 463 (59.1%) | Living alone  Mean MMSE: 19.8  Living with others  Mean MMSE: 19.7 | No detail of assessment or examination | 2001-2004  (3 years) | Hospitalisation, nursing home admission, weight loss, death | Carer and self-report | N/A | 0.27  351 (31.0%) in 2 years | Living alone  1.33 (1.01–1.74)  Home care services provided by non-healthcare professionals  1.40 (1.09– 1.80 | Age, sex, duration of disease (years), time since diagnosis (years), ADL score, number of non-dementia medications, antipsychotic medication, number of chronic diseases, homecare services used, income (euro/month), primary caregiver is female, primary caregiver is spouse |
| **Thorpe***  **2010** [30]  USA  Research cohort (NLCS) | 1,186  100% | 75.7  Male: 100% | No information | No detail of assessment of examination | 1997-1998  (1 year) | ACSC hospitalisations, non-ACSC hospitalisations | Hospital records | Not available | 434 (36.6%) in 1 year | County rurality  Rural 1.97 (1.08-3.58)  Micropolitan  1.06 (0.59-1.89)  Small metropolitan  0.87 (0.54-1.38)  Large metropolitan  Reference  Carer age in years  0.98 (0.96-1.00)  Carer ethnicity  White (Ref)  African American:  1.09 (0.60-1.96)  Relationship to PWD  Carer is wife (Ref)  Carer is other:  0.78 (0.39-1.59)  Carer education  1.00 (0.92-1.08)  Carer age  1.04 (1.01-1.08)  PWD characteristics  Age  1.04 (1.01-1.08)  ADL limitations  1.21 (1.14-1.30)  Behavioural disturbance  1.01 (0.99-1.03)  CCI:  1.18 (1.06-1.31)  Count of comorbid conditions  1.13 (0.99-1.31)  Enabling characteristics  Carer perceived financial adequacy  1.26 (0.86-1.85)  PWD health insurance  Any private or Medicaid:  0.72 (0.43-1.20)  Medicare only  0.92 (0.31-2.74)  Medicare + VA  0.74 (0.40-1.39)  VA only (Reference)  Carer instrumental social support  1.01 (0.98-1.03)  Carer emotional social support  1.03 (0.98-1.09)  Carer limitations from comorbidities  1.00 (0.95-1.05)  Carer depression  1.01 (0.95-1.07)  Primary care shortage area  No (Reference)  1.61 (1.00-2.57)  Community health clinic in county  No (Reference)  0.45 (0.28-0.70)  Distance to nearest VA medical center  1.00 (0.99-1.02) | County rurality, carer age, carer ethnicity, relationship to PWD, carer education in years, carer age  **PWD characteristics** age, sex, (ADL limitations, behavioural disturbance, number of comorbidities)  **Enabling characteristics** Carer perceived financial adequacy, carer health insurance, carer instrumental social support, carer emotional social support, carer limitations from comorbidities, carer depression  **County-level enabling indicators** (Primary care shortage area Y/N), Community health clinic in county (Y/N), Distance to nearest VA medical centre (miles), Hospitalisations (Any hospitalisation, Any ACSC hospitalisation, Any Non-ACSC  Hospitalisation) |
| **Tian**  **2013** [31]  USA  Insurance registers (MarketScans, Medicare) | 8,977 | PWD and dysphagia  81.1  N=485 (5.4%)  Female: 252 (52.0%)  PWD no dysphagia 81.0  N=8492 (94.6%)  Female: 246 (50.7%) | No information | No detail of assessment or examination. | 2006-2009  (3 years) | All-cause and AD-related health care costs, resource utilizations, outpatient, ER visits, hospitalisations | Hospital records | N/A | 0.23  1,804 (20.1%) in 1 year | Dysphagia  2.26 (1.70-2.99)  Without dysphagia (ref)  p=0.001 | Age, sex, health plan type, geographic region, study index year, comorbidities, medication burden, comorbidities in baseline year |
| **Tolppanen**  **2015** [32]  Finland  National register | 27,948 | 79.7  Female: 18,934 (67.8%) | No information | Standardized assessment and examination | 2005-2009  (4 years) | Number of hospitalisations, number of hospital days, outpatient visits, total costs related to visits | Hospital records | 1.25 (1.22-1.28) p=<0.001 | 1.17  23,317 (83.0%) in 4 years | N/A | Age, sex, geographic region, comorbidities |
| **Zhao**  **2008** [33]  USA  MEDSTAT MarketScan Medicare Supplemental and Coordination of Benefits (COB) Database | 25,109 | 80.1  Female: 61.6% | No information | No detail on assessment or examination | 2003-4  (1 year) | Health care costs and utilization | Hospital records | 1.55 (no CI) | 30% in 1 year | N/A | Age, sex, geographic region, months of health plan enrolment during 2004, illness burden, |
| **Zhu**  **2015** [34]  USA  Research cohort (WHICAP) | 1,766  (171) | 82.5  Female: 65.6% | Cognitive score  Mean: -1.27 | Standardized assessment and examination | 1999–2010  (3 years) | Hospitalisation, length of stay, Medicare expenditure for the stay, ACS admissions | Hospital records | 1.46 (1.24-1.94) | 67 (30.3%) in 1 year | Functional deficits  1.27 (p=<0.01)  Cognitive deficits  0.76 (p=<0.05) | Age, sex, ethnicity, years of education, comorbidities, depressive symptoms, living arrangement, death during the study period, follow-up year |

Key: AchEI – acetylcholinesterase inhibitors; ACS – ambulatory care sensitive; ACT – Adult Changes in Thought; AD – Alzheimer’s Disease; ADAS-Cog – Alzheimer’s Disease Assessment Scale – Cognitive; ADAMS – Ageing, Demographics and Memory Study; ADL – Activities of Daily Living Scale; BIMC – Blessed Information and Memory Concentration test; BPSD – behavioural and psychological symptoms of dementia; CCI – Charlson Comorbidity Index; CDR – Clinical Dementia Rating; CPS – Cognitive Performance Scale; CRIS – Clinical Record Interactive Search; DLB – Dementia with Lewy Bodies; DSM – Diagnostic and Statistical Manual of Mental Disorders; ED – Emergency Department; ER – Emergency Room; FTD – frontotemporal dementia; HR – Hazard ratop; ICD – International Classification of Diseases; ID – Intellectual Disability; IRR – Incidence rate ratio; LOS – length of stay; MADRC - Massachusetts Alzheimer’s Disease Research Center; mBI – modified Barthel Index; MDS – Minimum Data Set; MMSE – Mini Mental State Examination; MNA – Mini Nutritional Assessment; NINCDS-ADRDA – National Institute of Neurological and Communicative Disorders and Stroke and the Alzheimer’s Disease and Related Disorders Association; NLCS - National Longitudinal Caregiver Study; NPI – Neuropsychiatric Inventory; OASIS – Outcome And Assessment Information Set; OR – odds ratio; PDD – Parkinson’s Disease Dementia; PLASA – Plan de Soin et d’Aide dans la maladie d’Alzheimer; PSMS – Physical Self Maintenance Scale; PWD – person with dementia; ref – reference category; REAl-FR – French Network on Alzheimer’s Disease; SDRIR - Scottish Dementia Research Interest Register; SNAC - Swedish National Study on Aging and Care; VaD – vascular dementia; WHICAP - Washington Heights-Inwood Columbia Aging Project

Notes: *Unable to determine covariate adjustment for analysis of association of factors with hospital admission

## References

[1] Albert SM, Costa R, Merchant C, Small S, Jenders RA, Stern Y. Hospitalization and Alzheimer’s disease: results from a community-based study. J Gerontol A Biol Sci Med Sci 1999;54:M267-71.

[2] Aupperle PM, Coyne AC. Primary vs. subspecialty care: A structured follow-up of dementia patients and their caregivers. Am J Geriatr Psychiatry 2000;8:167–70. doi:10.1097/00019442-200005000-00012.

[3] Axmon A, Karlsson B, Ahlstrom G. Health care utilisation among older persons with intellectual disability and dementia: a registry study. J Intellect Disabil Res 2016;60:1165–77. doi:https://dx.doi.org/10.1111/jir.12338.

[4] Browne J, Edwards D, Rhodes K, Brimicombe D, Payne R. Association of comorbidity and health service usage among patients with dementia in the UK: a population-based study. BMJ Open 2017;7:e012546. doi:https://dx.doi.org/10.1136/bmjopen-2016-012546.

[5] Brüggenjürgen B, Andersohn F, Ezzat N, Lacey L, Willich S. Medical management, costs, and consequences of Alzheimer’s disease in Germany: an analysis of health claims data. J Med Econ 2015;18:466–73. doi:10.3111/13696998.2015.1014090.

[6] Bynum J, Rabins P, Weller W, Niefeld M, Anderson G, Wu A, et al. Illness , Medicare Expenditures , and Hospital Use. J Am Geriatr Soc 2004;52:187–94. doi:10.1111/j.1532-5415.2004.52054.x.

[7] Callahan C, Arling G, Tu W, Rosenman M, Counsell S, Stump T, et al. Transitions in care for older adults with and without dementia. J Am Geriatr Soc 2012;60:813–20. doi:https://dx.doi.org/10.1111/j.1532-5415.2012.03905.x.

[8] Chen L, Reed C, Happich M, Nyhuis A, Lenox-Smith A. Health care resource utilisation in primary care prior to and after a diagnosis of Alzheimer’s disease: a retrospective, matched case-control study in the United Kingdom. BMC Geriatr 2014;14:76. doi:https://dx.doi.org/10.1186/1471-2318-14-76.

[9] Chen YH, Ho CH, Huang CC, Hsu YW, Chen YC, Chen PJ, et al. Comparison of healthcare utilization and life-sustaining interventions between elderly patients with dementia and those with cancer near the end of life: A nationwide, population-based study in Taiwan. Geriatr Gerontol Int 2017;17:2545–51. doi:10.1111/ggi.13084.

[10] Cortes F, Nourhashémi F, Guérin O, Cantet C, Gillette-Guyonnet S, Andrieu S, et al. Prognosis of Alzheimer’s disease today: A two-year prospective study in 686 patients from the REAL-FR Study. Alzheimer’s Dement 2008;4:22–9. doi:10.1016/j.jalz.2007.10.018.

[11] Forma L, Rissanen P, Aaltonen M, Raitanen J, Jylhä M. Dementia as a determinant of social and health service use in the last two years of life 1996-2003. BMC Geriatr 2011;11:14. doi:10.1186/1471-2318-11-14.

[12] Hill J, Fillit H, Shah SN, Del Valle MC, Futterman R. Patterns of healthcare utilization and costs for vascular dementia in a community-dwelling population. J Alzheimer’s Dis 2005;8:43–50. doi:10.3233/JAD-2005-8105.

[13] Jennings LA, Laffan AM, Schlissel AC, Colligan E, Tan Z, Wenger NS, et al. Health Care Utilization and Cost Outcomes of a Comprehensive Dementia Care Program for Medicare Beneficiaries. JAMA Intern Med 2019;179:161–6. doi:10.1001/jamainternmed.2018.5579.

[14] Kedia S, Chavan P, Boop S, Yu X. Health Care Utilization Among Elderly Medicare Beneficiaries With Coexisting Dementia and Cancer. Gerontol Geriatr Med 2017;3:233372141668904. doi:10.1177/2333721416689042.

[15] Kunik M, Snow A, Molinari V, Menke T, Souchek J, Sullivan G, et al. Health Care Utilization in Dementia Patients With Psychiatric Comorbidity. Gerontologist 2003;43:86–91. doi:10.1093/geront/43.1.86.

[16] Malone DC, McLaughlin TP, Wahl PM, Leibman C, Arrighi HM, Cziraky MJ, et al. Burden of Alzheimer’s disease and association with negative health outcomes. Am J Manag Care 2009;15:481–8.

[17] Maust D, Kales H, McCammon R, Blow F, Leggett A, Langa K. Distress Associated with Dementia-Related Psychosis and Agitation in Relation to Healthcare Utilization and Costs. Am J Geriatr Psychiatry 2017;25:1074–82. doi:10.1016/j.jagp.2017.02.025.

[18] Miu D, Ying K, Hk M, Uk M, Cuhk MPH, Edin F, et al. Hospital admission risk in community dwelling elderly dementia subjects 2018;9:39–43. doi:10.24816/jcgg.2018.v9i2.02.

[19] Mueller C, Perera G, Rajkumar A, Bhattarai M, Price A, O’Brien J, et al. Hospitalization in people with dementia with Lewy bodies: Frequency, duration, and cost implications. Alzheimer’s Dement Diagnosis, Assess Dis Monit 2017;10:143–52. doi:10.1016/j.dadm.2017.12.001.

[20] Mueller C, Molokhia M, Perera G, Veronese N, Stubbs B, Shetty H, et al. Polypharmacy in people with dementia: Associations with adverse health outcomes. Exp Gerontol 2018;106:240–5. doi:10.1016/j.exger.2018.02.011.

[21] Nourhashemi F, Amouyal-Barkate K, Gillette-Guyonnet S, Cantet C, Vellas B. Living alone with Alzheimer’s disease: cross-sectional and longitudinal analysis in the REAL.FR Study. J Nutr Health Aging 2005;9:117–20.

[22] Phelan EA, Borson S, Grothaus L, Balch S, Larson EB. Association of incident dementia with hospitalizations. JAMA 2012;307:165–72. doi:https://dx.doi.org/10.1001/jama.2011.1964.

[23] Pimouguet C, Rizzuto D, Fastbom J, Lagergren M, Fratiglioni L, Xu W. Influence of Incipient Dementia on Hospitalization for Primary Care Sensitive Conditions: A Population-Based Cohort Study. J Alzheimers Dis 2016;52:213–22. doi:https://dx.doi.org/10.3233/JAD-150853.

[24] Rudolph J, Zanin N, Jones R, Marcantonio E, Fong T, Yang F, et al. Hospitalization in community-dwelling persons with Alzheimer’s disease: Frequency and causes. J Am Geriatr Soc 2010;58:1542–8. doi:10.1111/j.1532-5415.2010.02924.x.

[25] Russ T, Parra M, Lim A, Law E, Connelly P, Starr J. Prediction of general hospital admission in people with dementia: cohort study. Br J Psychiatry 2015;206:153–9. doi:https://dx.doi.org/10.1192/bjp.bp.113.137166.

[26] Sköldunger A, Fastbom J, Wimo A, Fratiglioni L, Johnell K. Impact of Inappropriate Drug Use on Hospitalizations, Mortality, and Costs in Older Persons and Persons with Dementia: Findings from the SNAC Study. Drugs and Aging 2015;32:671–8. doi:10.1007/s40266-015-0287-4.

[27] Sloane P, Schifeling C, Beeber A, Ward K, Reed D, Gwyther L, et al. New or Worsening Symptoms and Signs in Community-Dwelling Persons with Dementia: Incidence and Relation to Use of Acute Medical Services. J Am Geriatr Soc 2017;65:808–14. doi:https://dx.doi.org/10.1111/jgs.14672.

[28] Sommerlad A, Perera G, Mueller C, Singh-Manoux A, Lewis G, Stewart R, et al. Hospitalisation of people with dementia: evidence from English electronic health records from 2008 to 2016. Eur J Epidemiol 2019. doi:10.1007/s10654-019-00481-x.

[29] Soto M, Andrieu S, Gares V, Cesari M, Gillette-Guyonnet S, Cantet C, et al. Living alone with alzheimer’s disease and the risk of adverse outcomes: Results from the plan de soin et d’aide dans la maladie d’alzheimer study. J Am Geriatr Soc 2015;63:651–8. doi:10.1111/jgs.13347.

[30] Thorpe J, Van Houtven C, Sleath B, Thorpe C. Rural-urban differences in preventable hospitalizations among community-dwelling veterans with dementia. J Rural Health 2010;26:146–55. doi:https://dx.doi.org/10.1111/j.1748-0361.2010.00276.x.

[31] Tian H, Abouzaid S, Sabbagh M, Chen W, Gabriel S, Kahler K, et al. Health care utilization and costs among patients with AD with and without dysphagia. Alzheimer Dis Assoc Disord 2013;27:138–44. doi:https://dx.doi.org/10.1097/WAD.0b013e318258cd7d.

[32] Tolppanen A-M, Taipale H, Purmonen T, Koponen M, Soininen H, Hartikainen S. Hospital admissions, outpatient visits and healthcare costs of community-dwellers with Alzheimer’s disease. Alzheimers Dement 2015;11:955–63. doi:https://dx.doi.org/10.1016/j.jalz.2014.10.005.

[33] Zhao Y, Kuo T-C, Weir S, Kramer MS, Ash AS. Healthcare costs and utilization for Medicare beneficiaries with Alzheimer’s. BMC Health Serv Res 2008;8:108. doi:https://dx.doi.org/10.1186/1472-6963-8-108.

[34] Zhu C, Cosentino S, Ornstein K, Gu Y, Andrews H, Stern Y. Use and cost of hospitalization in dementia: longitudinal results from a community-based study. Int J Geriatr Psychiatry 2015;30:833–41. doi:https://dx.doi.org/10.1002/gps.4222.

## Table S3: Quality rating criteria and scores for included studies

**Table S3a: modified Newcastle Ottawa Quality Assessment Scale for assessing risk of bias**

Note: A study can be awarded a maximum of one star for each numbered item within the Selection and Outcomes categories. A maximum of two stars can be given for Comparability. Total score is out of six and studies with five or six stars are considered at low risk of bias.

**Selection**

1. Is the case definition of dementia adequate?
   1. yes, routine neuropsychological examination using validated diagnostic criteria e.g. DSM/ICD*
   2. yes, e.g. hospital records linkage or national databases
   3. self-reported
   4. no description or unclear
2. Representativeness of the cases
   1. consecutive or obviously representative cases *****
   2. potential for selection biases or not stated

**Comparability**

1. Comparability of cases and controls on the basis of the design or analysis
   1. study controls for age and sex *****
   2. study controls for ONLY age or ONLY sex
   3. study controls for comorbidities as well as age and sex *****
   4. no description or unclear

**Outcomes**

1. Ascertainment of outcome
   1. secure record (e.g. insurance/hospital records or national register) *****
   2. interview with person with dementia and an informant
   3. self-reported by informant
   4. self-reported by person with dementia or no description/unclear
2. Adequacy of follow up
   1. follow up data derived from national/hospital databases including all participants *****
   2. high follow up: 70-100% of subjects accounted for *****
   3. low follow up: 45-69% of subjects accounted for
   4. very low follow up: <45% of subjects accounted for and no description of those lost
   5. no description or unclear

**Table S3b: risk of bias ratings of 32 included papers**

|  | Quality criteria:  Total marks possible: | Selection  * | * | | Comparability  ** | Outcomes  * | * | | 6 | |  |
| --- | --- | --- | --- | --- | --- | --- | --- | --- | --- | --- | --- |
|  | **Author** | **1** | **2** | **3** | | **4** | | **5** | | **Total** | |
| **1** | Albert  1999 | * | * | ** | | N | | N | | 4 | |
| **2** | Aupperle  1999 | * | N | N | | N | | * | | 2 | |
| **3** | Axmon  2016 | N | * | * | | * | | * | | 4 | |
| **4** | Browne  2017 | N | * | * | | * | | * | | 4 | |
| **5** | Brüggenjürgen  2015 | N | * | ** | | * | | * | | 5 | |
| **6** | Bynum  2004 | N | * | * | | * | | * | | 4 | |
| **7** | Callahan  2012 | N | * | N | | * | | * | | 3 | |
| **8** | Chen  2014 | N | * | * | | * | | * | | 5 | |
| **9** | Chen  2017 | N | * | ** | | * | | * | | 5 | |
| **10** | Cortes  2008 | * | * |  | | N | | * | | 3 † | |
| **11** | Forma  2011 | N | * | ** | | * | | * | | 5 | |
| **12** | Hill  2005 | N | * |  | | * | | * | | 3 † | |
| **13** | Jennings  2019 | N | N | ** | | * | | * | | 4 | |
| **14** | Kedia  2017 | N | * | ** | | * | | * | | 5 | |
| **15** | Kunik  2003 | N | N | ** | | * | | * | | 4 | |
| **16** | Malone  2009 | N | N | ** | | * | | * | | 4 | |
| **17** | Maust  2017 | * | * | ** | | * | | * | | 6 | |
| **18** | Miu  2018 | N | N | ** | | * | | * | | 4 | |
| **19** | Mueller  2017 | N | * | ** | | * | | * | | 5 | |
| **20** | Mueller  2018 | N | * | ** | | * | | * | | 5 | |
| **21** | Nourhashemi  2005 | * | * |  | | N | | * | | 3 † | |
| **22** | Phelan  2012 | * | * | ** | | * | | * | | 6 | |
| **23** | Pimouguet  2016 | * | * | ** | | * | | * | | 6 | |
| **24** | Rudolph  2010 | * | N | ** | | N | | * | | 4 | |
| **25** | Russ  2015 | * | * | * | | * | | * | | 5 | |
| **26** | Sköldunger  2015 | * | * | ** | | * | | * | | 6 | |
| **27** | Sloane  2017 | N | N | N | | N | | * | | 1 | |
| **28** | Sommerlad  2019 | N | * | ** for risk factors  * for rate | | * | | * | | 5  4 | |
| **29** | Soto  2015 | N | N | ** | | N | | N | | 4 | |
| **30** | Thorpe  2010 | N | N | ** | | * | | * | | 4 | |
| **31** | Tian  2013 | N | * | ** | | * | | * | | 4 | |
| **32** | Tolppanen  2015 | N | * | ** | | * | | * | | 5 | |
| **33** | Zhao  2008 | N | N | * | | * | | * | | 3 | |
| **34** | Zhu  2015 | * | * | ** | | * | | * | | 6 | |

Notes: Greyed out box indicates criteria not applicable; N = mark not given; † Comparability criteria not relevant as studies report incidences or percentages of hospitalisations, not comparisons or associations

## Table S4: Risk of hospitalisation in people with dementia compared to people without dementia: full details of GRADE rating of evidence strength for risk factors

| **Author** | **Relative risk estimate**  **(95% confidence interval)** | **Individual paper quality considerations** | **Individual paper quality^*^** | **Overall quality considerations** | **Overall grade** |
| --- | --- | --- | --- | --- | --- |
| **Albert**  **1999** | 2.30 (1.10-4.60)^†^ |  | Moderate | + consistency  + magnitude of effect  + no evidence of publication bias (Begg’s test p=0.13) | High |
| **Axmon**  **2016** | 4.19 (1.88–9.32) | -imprecision  -indirectness | Very low |  |  |
| **Bynum**  **2004** | 3.68 (3.62–3.73) |  | Moderate |  |  |
| **Malone**  **2009** | 1.68 (1.51-1.86) |  | Moderate |  |  |
| **Mueller**  **2017** | DLB: 1.22 (1.06-1.39)  AD: 0.91 (0.84-0.99) |  | Moderate |  |  |
| **Phelan**  **2012** | 1.41 (1.23-1.61) |  | High |  |  |
| **Pimouguet**  **2016** | 1.08 (0.75-1.53) | -imprecision | Moderate |  |  |
| **Sommerlad**  **2019** | 2.06 (1.95, 2.18) ^**^ |  | Moderate |  |  |
| **Tolppanen**  **2015** | 1.25 (1.22-1.28) |  | High |  |  |
| **Zhu**  **2015** | 1.46 (1.24-1.94) | -imprecision | Moderate |  |  |
| **Zhao**  **2008** | 1.55 (no CI) |  | Moderate |  |  |
| **Studies of people with dementia at the end of life** | | | | | |
| **Chen**  **2017** | 1.14 (0.91–1.41) | -indirectness | Moderate | - inconsistency | Very low |
| **Forma**  **2011** | 0.33 (0.31-0.35) | -indirectness | Low |  |  |

Notes: * Paper quality based on risk of bias derived from Newcastle-Ottawa criteria rating and additional individual paper quality considerations; ^†^ Comparing people with severe dementia to people without dementia; **Refers to emergency hospital admissions

## Table S5: Percentage of study participants hospitalized in study period

| Author  Publication year | Country | Years of study (range) | Number (%) admitted during average length of study |
| --- | --- | --- | --- |
| Chen  2014 | England | 2008-10 | 416 (23.6%) in 0.5 years |
| Mueller  2017 | England | 2006-13 | 435 (46.3%) in 0.9 years |
| Kedia  2017 | USA | 2009 | 2,220 (25.9%) in 1 year |
| Maust  2017 | USA | 2002-08 | 217 (65.2%) in 1 year |
| Sommerlad  2019 | UK | 2008-16 | 5,127 (50.6%) in 1 year |
| Zhu  2015 | USA | 1999-2010 | 67 (30.3%) in 1 year |
| Russ  2015 | Scotland | Not reported | 274 (37.5%) in 1.2 years |
| Mueller  2018 | England | 2011-13 | 3,105 (66.5%) in 2 years |
| Phelan  2012 | USA | 1994-2007 | 427 (86.0%) in 3.5 years |
| Tolppanen  2015 | Finland | 2005-09 | 23,317 (83.0%) in 4 years |
| Aupperle  1999 | USA | 1997 | 16 (28.1%) in 0.5 years |
| Axmon  2016 | Sweden | 2002-11 | 32 (44.0%) in 1 year |
| Cortes  2008 | France | Not reported | 91 (26.1%) in 1 year |
| Nourhashemi  2005 | France | Not reported | 154 (26.8%) in 1 year |
| Thorpe  2010 | USA | 1997-98 | 434 (36.6%) in 1 year |
| Tian  2013 | USA | 2006-10 | 1,804 (20.1%) in 1 year |
| Zhao  2008 | USA | 2003-04 | 7,533 (30%) in 1 year |
| Albert  1999 | USA | 1996-97 | 70 (17.5%) in 1.75 years |
| Kunik  2003 | USA | 1997-99 | 356 (41.2%) in 1.8 years |
| Soto  2015 | France | Not reported | 351 (31.0%) in 2 years |
| Rudolph  2010 | USA | 1991-2006 | 542 (66.0%) in 2.2 years |
| Callahan  2012 | USA | 2001-4 | 399 (76.2%) in 4.7 years |
| Malone  2009 | USA | 2000-06 | 1338 (24.8%) in 5 years |
| Author  Publication year | Country | Years of study (range) | Number (%) admitted during average length of study |
| Chen  2017 | Taiwan | 2002-11 | 748 (82.3%) in the last year of life |
| Forma  2011 | Finland | 1998-2003 | 22,011 (64.3%) in the last 2 years of life |

Notes: Bold outline indicates studies with the lowest risk of bias (studies examining end of life are considered separately)

## Table S6: Association of potential risk factors with hospitalisation in people with dementia: full details of GRADE rating of evidence strength for risk factors

| **Factor** | **Categorisation** | **Study** | **Adjusted estimates** | **Individual paper quality considerations** | **Individual paper quality** | **Overall quality considerations** | **Confidence in evidence** |
| --- | --- | --- | --- | --- | --- | --- | --- |
| **Person with dementia – demographics** | | | | | | | |
| Age | Higher age | Sköldunger 2015 | 1.01 (0.97–1.05) | - imprecision | Moderate | + consistency  + No evidence of publication bias  (Begg’s test p=0.60) | High |
|  |  | Thorpe 2010* | 1.04 (1.01-1.08) |  | Moderate |  |  |
|  |  | Sommerlad 2019** | 1.03 (1.02, 1.03) |  | Moderate |  |  |
|  |  | Malone 2009 | 1.02 (1.01-1.02) |  | Moderate |  |  |
|  | Per 5 years older | Russ 2015 | 1.07 (0.99–1.16) |  | High |  |  |
|  | ≥75 years (<75 years = ref) | Rudolph 2010 | 1.51 (1.26–1.81) |  | Moderate |  |  |
|  | ≥90 years (<75 years = ref) | Browne 2017 | 1.66 (1.41-1.95) | + dose response | Moderate |  |  |
| Male Sex | (Female = ref) | Russ 2015 | 1.33 (1.04-1.69) |  | High | - inconsistency + magnitude of effect  + No evidence of publication bias (Beggs test p=0.62) | Low |
|  |  | Rudolph 2010 | 1.27 (1.04-1.54) |  | Moderate |  |  |
|  |  | Sköldunger 2015 | 1.14 (0.58-2.22) | - imprecision | Moderate |  |  |
|  |  | Browne 2017 | 1.19 (1.08-1.29) |  | Moderate |  |  |
|  |  | Sommerlad 2019** | 1.30 (1.19, 1.41) |  | Moderate |  |  |
|  |  | Malone 2009 | 0.86 (0.77-0.95) |  | Moderate |  |  |
| Minority ethnicity | White = ref | Rudolph 2010 | 0.84 (0.58–1.22) |  | Moderate | + magnitude of effect | Low |
|  |  | Sommerlad 2019** | 0.79 (0.72-0.86) |  | Moderate |  |  |
| Less education | ≥12 years = ref | Rudolph 2010 | 1.18 (0.92–1.52) |  | Moderate | + consistency | Low |
|  |  | Sköldunger 2015 | 1.11 (0.63-1.81) | - imprecision | Moderate |  |  |
| Socioeconomic status | Per decile increase in deprivation | Sommerlad 2019** | 1.05 (1.01-1.09) |  | Moderate |  | Very low |
| Being unmarried | Married = ref | Rudolph 2010 | 0.92 (0.75–1.13) |  | Moderate | - inconsistency | Very low |
|  |  | Sommerlad 2019** | 1.11 (1.02-1.21) |  | Moderate |  |  |
| **Person with dementia - other health conditions** | | | | | | | |
| Presence of physical comorbidities | Any comorbidity  (No co-morbidities = ref) | Sköldunger 2015 | 1.47 (0.89–2.63) | - imprecision | Moderate | + consistency  + magnitude of effect  + No evidence of publication bias  (Beggs test p=0.60) | Moderate |
|  |  | Russ 2015 | 1.28 (1.00–1.65) |  | High |  |  |
|  |  | Rudolph 2010 | 1.87 (1.57–2.23) |  | Moderate |  |  |
|  | Problem with physical illness | Sommerlad 2019** | 1.73 (1.59-1.88) |  | Moderate |  |  |
|  | CCI score | Thorpe 2010* | 1.18 (1.06-1.31) |  | Moderate |  |  |
|  | 0 or 1 comorbidities  ≥6 comorbidities  (2 or 3 comorbidities = ref) | Browne 2017 | 0.88 (0.78-0.99)  1.62 (1.44-1.83) | + evidence of dose response | Moderate |  |  |
|  | No dysphagia = ref | Tian 2013 | 2.26 (1.70-2.99) | - indirectness | Moderate |  |  |
| Presence of psychiatric comorbidities | Total NPI score (per SD increase)  Agitation (per SD increase) | Russ 2015 | 1.21 (1.08–1.36)  1.28 (1.14–1.43) |  | High | - inconsistency | Low evidence of no association |
|  | No behavioural disturbance = ref | Maust 2017 | 0.63 (0.36-1.09) | - imprecision | Moderate |  |  |
|  |  | Thorpe 2010* | 1.01 (0.99-1.03) |  | Moderate |  |  |
|  | No behavioural disturbance = ref | Albert 1999 | No significant differences |  | Moderate |  |  |
|  | Agitated behaviour  Self-injury  Problem drink/drugs  Hallucinations  Depressed mood | Sommerlad 2019** | 1.02 (0.92-1.13)  1.24 (0.92-1.68)  1.12 (0.91, 1.38)  1.03 (0.92, 1.15)  1.14 (1.02, 1.26) |  | Moderate |  |  |
| Previous acute hospitalisation | No previous hospitalisation = ref | Rudolph 2010 | 1.65 (1.37–1.99) |  | Moderate | + magnitude of effect | Low |
| Use of medications | ≥7 medications (0-3 = ref) | Mueller 2018 | 1.32 (1.19–1.47) |  | High | + magnitude of effect | Moderate |
|  | No inappropriate drug use = ref | Sköldunger 2015 | 1.88 (1.03–3.43) | - imprecision | Moderate | - inconsistency  + magnitude of effect | Low |
|  |  | Russ 2015 | 1.24 (0.82–1.88) |  | High |  |  |
|  | Prescribed antipsychotics | Russ 2015 | 1.32 (0.77–2.27) |  | High |  | Low |
| Presence of any ID | Unplanned admission | Axmon 2016 | 1.90 (1.02–3.55) | - imprecision  - indirectness | Very Low |  | Very Low |
| Lower level of functional ability | IADL score (per SD increase)  PSMS score (per SD increase) | Russ 2015 | 1.08 (0.95–1.22)  1.18 (1.04-1.33) |  | High | + consistency  + magnitude of effect | Moderate |
|  | Independent = ref | Sköldunger 2015 | 1.19 (0.61–2.30) | - imprecision | Moderate |  |  |
|  | ADL limitations | Zhu 2015 | 1.27 (p=<0.01) | - imprecision | Moderate |  |  |
|  |  | Thorpe 2010* | 1.21 (1.14-1.30) |  | Moderate |  |  |
|  | Problem with daily living | Sommerlad 2019 ** | 1.18 (1.08, 1.28) |  | Moderate |  |  |
| **Person with dementia – lifestyle factors** | | | | | | | |
| Living arrangements | Living in a care home  Community dwelling = ref | Sköldunger 2015 | 0.26 (0.13–0.50) | - imprecision | Moderate | + magnitude of effect | Low |
|  | Living alone  (Living with others = ref) | Soto 2015 | 1.33 (1.01–1.74) |  | Low | - inconsistency | Very Low |
|  |  | Nourhashemi 2005 | No significant differences |  | Moderate |  |  |
| Neighbourhood characteristics | Rural (metropolitan=ref)  Primary care shortage (No shortage = ref) | Thorpe 2010* | 1.97 (1.08-3.58)  1.61 (1.00-2.57) |  | Moderate | + magnitude of effect | Low |
| **Person with dementia – characteristics of the dementia** | | | | | | | |
| More severe dementia | DSR score >=2 points  BIMC score >=15 points | Rudolph 2010 | 1.20 (0.98–1.47)  0.99 (0.79-1.24) |  | Moderate | + consistency | Moderate evidence of no association |
|  | MMSE score (per SD increase)  CDR score  0 or 0.5 = ref  2 or 3  CDR sum of values | Russ 2015 | 1.00 (0.88–1.14)  1  1.24 (0.89–1.72)  1.04 (0.92–1.17) |  | High |  |  |
|  | Cognitive deficits in 5 domains† | Zhu 2015 | 0.76 (p=<0.10) | - imprecision | Moderate |  |  |
|  | MMSE (per unit decrease) | Sommerlad 2019 ** | 1.01 (1.00-1.01) |  | Moderate |  |  |
| Non-AD dementia | DLB vs AD | Mueller 2017 | 1.46 (1.10-1.94) |  | High | + magnitude of effect | Low |
|  | Non-AD dementia vs AD | Russ 2015 | 1.16 (0.90-1.49) |  | High | + consistency | Low |
|  |  | Sommerlad 2019 ** | 1.38 (1.28-1.50) |  | Moderate |  |  |
| **Person with dementia – healthcare services use** | | | | | | | |
| Having health insurance | VA only = ref | Thorpe 2010* | 0.72 (0.43-1.20) |  | Moderate |  | Very low |
| Receiving dementia care program | Standard care = ref | Jennings 2019 | No significant differences |  | Moderate |  | Very low |
| Homecare provided by non-professionals | Provided by professionals = ref | Soto 2015 | 1.40 (1.09–1.80) |  | Moderate | + magnitude of effect | Low |
| **Caregiver – demographics and characteristics** | | | | | | | |
| High carer distress due to neuropsychiatric symptoms | No neuropsychiatric symptoms = ref | Maust 2017 | 2.78 (1.73–4.46) | - imprecision | Moderate | + magnitude of effect | Low |
| Having a carer | No carer = ref | Russ 2015 | 1.03 (0.64–1.64) |  | High |  | Low |
| Caregiver characteristics | Older age | Thorpe 2010* | 0.98 (0.96-1.00) |  | Moderate |  | Very Low |
|  | Minority ethnicity |  | 1.09 (0.60-1.96) |  |  |  |  |
|  | Education (years) |  | 1.00 (0.92-1.08) |  |  |  |  |
|  | Perceived financial adequacy |  | 1.26 (0.86-1.85) |  |  |  |  |
|  | Limitations from comorbidities |  | 1.00 (0.95-1.05) |  |  |  |  |
|  | CES-D depression score |  | 1.01 (0.95-1.07) |  |  |  |  |
|  | Instrumental social support |  | 1.01 (0.98-1.03) |  |  |  |  |
|  | Emotional social support |  | 1.03 (0.98-1.09) |  |  |  |  |
|  | Carer is wife = ref  Carer is daughter/sister |  | 1  0.78 (0.39-1.59) |  |  |  |  |

Key: AD - Alzheimer’s Disease; ADL – activities of daily living; BIMC - Blessed Information Memory Concentration scale; BPSD – behavioural and psychological symptoms of dementia; CDR - Clinical Dementia Rating, CES-D - Centre for Epidemiological Studies Depression scale; CI - confidence interval; DC - Deyo-Charlson scale for measuring comorbidities; DLB - Dementia with Lewy Bodies; DSR - Dementia Severity Rating; IADL - Instrumental Activities of Daily Living scale; ID – intellectual disability; NPI – Neuropsychiatric Inventory; PSMS - Physical self-maintenance scale; VA - Veteran’s Administration; Ref - reference; OR - odds ratio; HR - hazard ratio

Notes: * Figures relate to Ambulatory-care sensitive conditions; ** Figures relate to emergency admissions; † Author’s own assessment scale, measured cognition according to memory, abstract reasoning, visual-spatial, language and executive speed; full data on covariate adjustment in data extraction table (*Table S2*); (-) indicates strength of evidence downgraded due to listed factor, (+) indicates strength of evidence upgraded due to listed factor. Factors affecting strength of evidence are: consistency (unexplained heterogeneity of results or wide variance of point estimates across studies); directness (the generalisability of the study populations); imprecision (downgraded if number of study participants <349 or wide confidence intervals around the effect); magnitude of effect; presence of dose-response relationship and evidence of publication bias; bold indicates significant results in the adjusted estimates column
