# Supplementary material for: Hospitalisation rates and predictors in people with dementia: a systematic review and meta-analysis
Source: BMC Med. 2019 Jul 15;17:130. doi: 10.1186/s12916-019-1369-7 (PMC6628507; doi:10.1186/s12916-019-1369-7)
Supplement: Supplementary file 2 — Study protocol. (PDF 100 kb) [file 12916_2019_1369_MOESM2_ESM.pdf]

Hospitalisation of people with dementia: a systematic review of the rate and risk factors for general hospital admission

Hilary Shepherd, Andrew Sommerlad, Gill Livingston

Citation

Hilary Shepherd, Andrew Sommerlad, Gill Livingston. Hospitalisation of people with dementia: a systematic review of the rate and risk factors for general hospital admission. PROSPERO 2018 CRD42018091722 Available from:
http://www.crd.york.ac.uk/PROSPERO/display_record.php?ID=CRD42018091722

Review question

This review will aim to explore the following through searching the available literature:

A. What is the rate of general (non-psychiatric) hospital admission of people with dementia?

B. Are there any demographic and clinical predictors of hospital admission? This may include but is not limited to features such as age, sex, country of residence, socioeconomic status, date of dementia diagnosis, dementia type, health behaviours (i.e. smoking, alcohol and drug consumption), existing comorbidities (including both acute illnesses and chronic conditions), neuropsychiatric symptoms of dementia.

Searches

MEDLINE, EMBASE and PsycINFO will be accessed and searched using keywords such as, but not limited to:

Population

Adult* OR people with dementia OR person with dementia OR living with dementia

Exposure

Dement* OR Alzheimer* Disease OR Frontotemporal OR Lewy Bod*

Comparator

N/a

Outcome

Hospitali#ation OR patient admission

Study Design
Case control OR longitudinal OR cohort OR cross-sectional

Government reports, dissertations and other grey literature will be included in the Ovid search.

In addition, the reference lists of included papers will also be hand searched to ensure that all relevant papers are included.

No date or language restrictions will be applied.

Types of study to be included
Observational studies including cohort, case-control and cross-sectional studies.

As no previous systematic review has been carried out in this area, studies will be considered from all geographical locations across the world, and no date or language restraints will be applied in the search, to ensure all relevant studies are included.

Condition or domain being studied
Dementia, as defined in DSM, ICD, or equivalent, presenting as “significant cognitive decline from previous level of performance in one of more cognitive domains, which interferes with activities of daily living, and cannot be explained by delirium or another mental disorder” (American Psychiatric Association, 2013).

Any subtype of dementia will be included in this review, for example frontotemporal dementia, Alzheimer’s disease, dementia with Lewy bodies, etc.

Participants/population
Clinical or population cohorts of people with dementia of any age.

Intervention(s), exposure(s)
The exposures of interest are those relating to the demographic or clinical characteristics of the person with dementia. We aim to identify which factors are associated with the risk of hospital admission.

Comparator(s)/control
Not applicable.

Context

Main outcome(s)
Rate of hospital admissions after diagnosis of dementia (per person year) and how this compares to people without dementia

Additional outcome(s)
Secondary outcomes are risk factors associated with first hospital admission in people with dementia (for instance socioeconomic factors and demographic factors)

Data extraction (selection and coding)
The titles and abstracts of the references retrieved during the searches will be reviewed and those which apparently meet the inclusion criteria will be identified. The full texts of these papers will then be independently reviewed by two researchers, and decisions made regarding eligibility. The numbers of papers included and excluded will be detailed using a PRISMA flow diagram, and any disagreements will be resolved by discussion with a third researcher.

Data from the relevant papers will be extracted onto a pilot-tested form, and will include:

Study type;

Numbers of participants recruited for study;

Number of participants at follow up (including loss to attrition);

Average length of follow-up;

Country, year, author and setting of study, i.e. GP, memory or psychiatry clinic;

Definition of population sample, i.e. with Down Syndrome, over 65 years old;

Setting of dementia diagnosis, i.e. memory clinic;

Average severity of dementia, i.e. with MMSE score (including definition of severity);

Method of dementia diagnosis (i.e. DSM/ICD);

Covariates assessed and method of assessment: age, ethnicity, socioeconomic status, sex, dementia type and severity, neuropsychiatric symptoms of dementia, physical comorbidity, other relevant factors;

Primary outcomes measured;

Secondary outcomes measured;

Rate of hospital admission;

Factors associated with hospital admission;

Quality rating of study

Risk of bias (quality) assessment

The Newcastle-Ottawa Scale (NOS) will be used to assess the quality of each included paper. We will assess the effects of study quality on the findings and will consider them in the review discussion. Any missing data will be requested directly from study authors.

Strategy for data synthesis

Papers will be summarised with narrative synthesis. In addition, if the measurements of covariates are comparable, relative risk data will be pooled using a random effects meta-analysis, and we will consider the use of meta-regression to examine the effects of study design factors on findings.

Analysis of subgroups or subsets

Data may be analysed separately if trends are found, e.g., by age, risk factor, or demographic feature.

Contact details for further information

Hilary Shepherd
uctvhls@ucl.ac.uk

Organisational affiliation of the review

UCL
www.ucl.ac.uk

Review team members and their organisational affiliations

Ms Hilary Shepherd. UCL
Dr Andrew Sommerlad. UCL
Professor Gill Livingston. UCL

Anticipated or actual start date

02 April 2018

Anticipated completion date

03 September 2018

Funding sources/sponsors

MSc funding for Hilary Shepherd is through the UCL MSc in Dementia Scholarship

Conflicts of interest

None specified.

Language

English

Country

England

Stage of review

Review_Ongoing

Subject index terms status
Subject indexing assigned by CRD

Subject index terms
Dementia; Hospitalization; Hospitals, General; Humans; Patient Care; Risk Factors

Date of registration in PROSPERO
27 March 2018

Date of publication of this version
16 August 2018

Details of any existing review of the same topic by the same authors

Stage of review at time of this submission

Stage	Started	Completed
Preliminary searches	Yes	No
Piloting of the study selection process	Yes	No
Formal screening of search results against eligibility criteria	Yes	No
Data extraction	No	No
Risk of bias (quality) assessment	No	No
Data analysis	No	No

Versions
27 March 2018
16 August 2018

PROSPERO
This information has been provided by the named contact for this review. CRD has accepted this information in good faith and registered the review in PROSPERO. CRD bears no responsibility or liability for the content of this registration record, any associated files or external websites.
